# Psychotherapeutic Care in Austria 2026–2040: An FTE-Calibrated Simulation of Workforce, Funding and Care Capacity

**DOI:** 10.3390/healthcare14213610

**Published:** 2026-10-25

**Authors:** Markus Boeckle, Barbara Haid, Fabienne Patek, Henriette Löffler-Stastka

**Affiliations:** 1 Department of Science and Research, Austrian Federal Association for Psychotherapy (ÖBVP), Löwengasse 3/3/Top 4, 1030 Vienna, Austria; oebvp.haid@psychotherapie.at (B.H.); oebvp.patek@psychotherapie.at (F.P.); 2 Department of Psychiatry and Psychotherapeutic Medicine, University Hospital Tulln—NOE LGA, Karl Landsteiner University, Alter Ziegelweg 10, 3430 Tulln, Austria; 3 Department of Psychoanalysis and Psychotherapy and Postgraduate Unit, Medical University of Vienna, Spitalgasse 23, 1090 Vienna, Austria; henriette.loeffler-stastka@meduniwien.ac.at

**Keywords:** psychotherapy care, workforce planning, full-time-equivalent modeling, Monte Carlo simulation, Psychotherapy Act 2024, health economics, access to care, treatment intensity, care gap, Austria

## Abstract

Background/Objectives: Psychotherapeutic care in Austria faces the transition introduced by the Psychotherapy Act 2024 (PThG 2024), an aging profession, and a need far exceeding publicly funded provision (potential capacity ≈669,000 vs. ≈199,250 publicly (co-)funded patients per year in 2022). Methods: We developed an FTE-calibrated Monte Carlo simulation (30,000 iterations per scenario, 2026–2040; anchors: 21 treatment hours/week, 13 units/patient/year) distinguishing potential, accessible, and fully funded capacity while treating supervised trainees as a separate, survey-anchored capacity line whose capacity rises with insurance funding, including the PThG 2024 transition (master’s entry 2026/27; the old pathway ends in 2038), a static robustness matrix at the 2023 capacity anchor (need 5–12%; intensity 13–40 units/patient/year) and weekly-hours-anchor sensitivities (21/15.8/13.3 h/week). Results: At 13 units/patient/year, the expansion scenario closes the median care gap from 2028 and the partial-funding scenario from 2032, largely by funding the services of trainees who are already treating under supervision; at 30 units/patient/year, even a full expansion covers only ≈30% of a 9% need at the 2023 anchor (≈49% with projected 2040 capacity); and under lower weekly-hours anchors, the gap closes later or not at all. The 2038 end of the old pathway produces a workforce-growth cliff: sustaining growth requires about 1500 master’s places per year with matching phase-3 places. Population needs dominate cost uncertainty (Spearman ρ up to 0.79) ahead of the activation rate; exploratory cumulative follow-on costs up to 2040 approximate €83/13/1 billion (A/B/C). Conclusions: Activating existing capacity—including trainees’ services, whose capacity line rests on a single unweighted survey and expert assumptions—is the dominant short-term lever; master’s and phase-3 places determine workforce growth after 2038; and in higher-intensity treatment scenarios, capacity expansion becomes indispensable.

## 1. Introduction

Psychotherapeutic care in Austria is at a structural turning point, where three developments overlap. First, the Psychotherapy Act 2024 (PThG 2024) fundamentally transforms professional training into a consecutive bachelor’s–master’s system followed by a specialist training phase [1]. Second, the existing profession is aging: of the 11,676 psychotherapists registered in the professional register in 2023, 27% were already over 65 years old [2]. Third—and this has been most clearly underestimated in the debate so far—the computed personnel capacity exceeds publicly funded care several times over.

Specifically, recent analyses [2,3] have shown that, at an empirical median of 21 treatment hours per week and 13 treatment units per patient per year, the theoretical total capacity is roughly 669,000 persons per year. In 2022, however, only about 199,250 persons received partially or fully publicly funded psychotherapy, while only about 96,500 persons received fully funded psychotherapy [2,3]. Against an estimated need or treatment willingness of 9% of the population (~805,000 persons in the insured reference population used throughout this model [4]), the gap between computed capacity and socially accessible care becomes evident.

This implies a fundamental conceptual shift: the central challenge of Austrian psychotherapeutic care is not primarily a workforce or recruitment problem but a funding, access, and structural problem. While reform-cohort models have traditionally focused on the number of licensed therapists, the present FTE-calibrated model shows that even a growing profession cannot provide the needed coverage of care without corresponding funding activation.

The health-economic relevance of this diagnosis is considerable. International evidence indicates that longer waiting times for psychotherapeutic treatment are associated with poorer treatment outcomes and with higher economic costs of care, although effect sizes vary across settings [5,6]. Chronic mental disorders in turn cause productivity losses, sick leave, and early retirement on an economically substantial scale [7,8]. Persons in training under supervision (old system: in Ausbildung unter Supervision, i.A.u.S.; new system: Psychotherapeut:innen in Fachausbildung unter Lehrsupervision, PiFALS; henceforth ‘supervised trainees’)—around 2799 in 2023 [9] and about 3200 in 2025 (Section 2.3)—occupy a critical dual function: they are part of both the current care capacity and the future workforce reserve. Their services are currently not funded by statutory health insurance, so their funding, or lack thereof, acts on both dimensions simultaneously.

This paper integrates these empirical anchors for the first time into a consistent simulation model that renders the structural sensitivities of Austrian psychotherapeutic care up to 2040 transparent, differentiates three capacity levels—potential, accessible/partially funded, and fully funded—and models supervised trainees of the old and new training systems as a capacity line of their own.

## 2. Background: The Austrian Care Situation

### 2.1. The Profession and the FTE Anchor

As of 2023, 11,676 psychotherapists were registered in the Austrian professional register [2,9]. Of these, 8523 (73%) were under 65 years of age and 3159 (27%) were over 65. The study by Dinhof et al. [2] provided the first empirically robust data on actual care activity: the median of weekly treatment hours was 21, which, at 45 working weeks per year, yielded an annual treatment capacity of 945 h per full-time-equivalent therapist. At the current value of 13 units per patient per year [2], this corresponds to a computed capacity of 72.7 persons per therapist per year; the parallel social-insurance accounting reports an average of 13.8 units across all funding channels in 2022 [3].

This FTE calibration is methodologically central because it shifts the model basis from headcount logic (‘how many therapists are registered?’) to capacity logic (‘how many persons can actually be treated?’). The underlying 13 treatment units per person per year reflect the current status quo. A systematic review of dose–response effects in routinely delivered psychological therapies reports optimal doses between four and 26 sessions, which varies with setting, clinical population, and outcome criterion, with 11 to 19 sessions typically needed for both reliable and clinically significant improvement [10]; depending on the clinical picture, 30 to 40 units per year serve as higher-intensity reference scenarios—adequate intensity varies substantially with the diagnosis, severity, treatment modality, and treatment goals. The model thus maps a best-case theoretical capacity at the current treatment intensity, and adjusting to higher treatment intensities reduces the theoretical capacity correspondingly.

### 2.2. Care Situation and the Funding Gap

The care figures for the reference year 2022 result from the funding channels reported by Dinhof et al. [2]: about 95,100 persons received fully funded psychotherapy through care associations and institutions and about 1400 through the ÖGK’s own psychotherapeutic facilities—combined together as ≈96,500 fully funded persons (≈1.1% of the total population)—while a further ≈102,750 persons received subsidized psychotherapy in private practice. Together, these three channels reach ≈199,250 persons through partially or fully funded psychotherapy in 2022, which Dinhof et al. [2] also reported as a minimum figure. This quantity is not identical to the 370,389 persons counted under the broader psychosocial care definition of the social-insurance analysis [3] and must not be substituted for it. This contrasts with a treatment willingness and epidemiological need of about 9% of the population [4]. Because publicly funded psychotherapy is an insurance benefit, all model calculations refer to the 8,942,791 persons entitled to benefits in Austrian social health insurance in the 2022 annual average [11] rather than to the resident population of about 9.1 million; at 9%, this corresponds to an absolute need of roughly 805,000 persons per year (≈820,000 if referred to the resident population).

The difference between the computed capacity (669,000) and fully funded care (96,500) illustrates the scale of the funding gap: 86% of the potential psychotherapeutic capacity is not translated into socially accessible, fully funded care. Even when including partially funded services, care reaches only about 30% of potential capacity and only about 24% of epidemiological need. This discrepancy is the central structural finding that the present model attempts to quantify and project.

### 2.3. Training Structure and the Psychotherapy Act 2024

The PThG 2024 fundamentally replaces the Psychotherapy Act of 1990. In the new system, training is structured in three consecutive phases: (1) a six-semester bachelor’s program in psychotherapy, (2) a four-semester master’s program, and (3) a subsequent phase of specialist training under teaching supervision (phase 3) [1,12]. Full licensure occurs only after completion of all three phases. Neither the PThG 2024 nor the PTh-AAQV 2024 fixes a minimum duration for phase 3 in calendar years; both specify minimum contents and volumes instead (at least 2050 training hours in total, **of which** 1000 h of psychotherapeutic treatment, 400 h of theory, 200 h of teaching supervision and 200 h of self-experience) [1,12]. The effective duration of phase 3 is therefore not a legal constant but a model parameter, and the simulation varies this accordingly (Section 3.9).

In the old system, 5021 persons were still enrolled in the Fachspezifikum (method-specific training) in 2023 [13], and 2799 persons were undergoing specialist training with teaching supervision [9]; completions in the old system reached 514 in 2023 [13]. The most recent training statistics (reference date 1 June 2025) show that the old pathway is still expanding ahead of its statutory closure: 5601 persons were enrolled in the Fachspezifikum, with 929 admissions, 624 completions, and 91 drop-outs in the 2024/25 reporting year; 3238 of the enrolled persons (57.8%) already held the status ‘in training under supervision’ (i.A.u.S.), which entitles them to treat patients under supervision, meaning that—as the training statistics note—they already contribute to psychotherapeutic care, although their services are not refundable by statutory health insurance [14]. The transitional provisions of the PThG 2024 fix the horizon of this pathway: admission to the old Fachspezifikum is inadmissible after 1 October 2030, and old-pathway training must be completed by 30 September 2038 (§§ 60(4) and 61(3) PThG 2024) [1]. Old-system completions will therefore end in 2038. In the new system, the master’s programs in psychotherapy are offered from the winter semester 2026/27; persons with an equivalent bachelor’s degree enter at the master’s level (about 500 places per year at public universities, a planning figure of the Federal Ministry communicated to the authors; and an estimated 500 at private universities); and specified professions—among which including psychiatrists and other physicians with a qualification in psychotherapeutic medicine, clinical and health psychologists, and music therapists—may enter phase 3 directly (§ 10 PThG 2024) [1,14]. Two provisions establish trainees as a policy lever from the outset: from 1 October 2026, persons in training under supervision are listed in the professional register with the addition ‘in specialist training under teaching supervision’, which makes them visible as providers of care, although the listing alone does not make their services an insurance benefit—§ 135(1)(3) of the General Social Insurance Act (ASVG, as amended by BGBl. I No. 145/2024) covers psychotherapy only by persons entitled to independent practice under § 22 PThG 2024 [1]; and the first licensures from the master’s route are expected from about 2030–2032, several years earlier than the first graduates of the full bachelor’s–master’s route.

### 2.4. Demographic Development of the Profession

The profession’s age structure points to a substantial retirement wave over the next 10 to 15 years. Therapists over 65 years old (3159 persons) continue to contribute to care capacity albeit with reduced weekly hours [2]. The model assumes a retirement/exit rate of about 3.5% per year (range: 2–5.5%), yielding a modeled exit figure of about 420 persons per year in 2026, rising with the growing stock to 832 persons per year in 2040 under Scenario A and 937 under Scenario C.

## 3. Materials and Methods

### 3.1. Theoretical Framework

The model operates on five interlinked analytical levels. **Level 1—Training policy:** Decisions on training architecture, phase-3 capacities, and funding act on the future workforce with a time lag; the latency between a training decision and its licensure effect is modeled for the master’s-entry route of the new system as two years of master’s studies plus a variable phase-3 duration of two to five years (mode three), so that the first licensures arrive about four to seven years after entry—because phase 3 carries no statutory minimum length, this latency is drawn from a distribution instead of being fixed (Section 3.9). Trainees in phase 3 already treat patients under teaching supervision, so the care effect of a training decision precedes its licensure effect. **Level 2—Workforce dynamics (FTE):** The stock dynamics of licensed therapists, calibrated with the empirical FTE anchor of 945 treatment hours/year and the age-corrected value of 57.31 persons per registered therapist (72.69 per fully active therapist), define the potential care capacity. **Level 3—Funding activation:** The share of potential capacity translated into accessible (partially/fully funded) care depends on care structures, tariffs, statutory-insurance contracting, and the institutional integration of teaching supervision; this activation rate is the genuinely policy-sensitive lever. **Level 4—Waiting time and chronification:** The care gap (need minus accessible capacity) determines chronification risk via waiting times; this relationship is qualitatively documented but not empirically quantified in precise risk curves by waiting duration, so the model treats it as a sensitivity parameter in three variants. **Level 5—Societal follow-on costs:** Chronification, sick leave, and productivity losses generate monetizable follow-on costs that quantify the economic value of investing in accessible care.

The model’s central theoretical thesis is that funding activation—the share of potential FTE capacity converted into socially accessible care—is the dominant health-policy lever. The number of licensed psychotherapists is a necessary but not sufficient condition to cover the care needed. A policy focused exclusively on training numbers risks leaving already existing potential unactivated.

### 3.2. Study Design

This is a deterministic–stochastic simulation model with probabilistic uncertainty analysis (Monte Carlo simulation, 30,000 iterations per scenario). The model is not a predictive forecasting model but a scenario model that represents structural sensitivities and ranges of possible developments under defined assumptions. The model logic follows discrete annual updates from 2026 to 2040. The source code is provided as Codes S1 (main model) and S2 (robustness re-calculation) and the documentation as Supplementary Materials S1–S3; the outputs are provided as Tables S1 and S2 (robustness matrix), Table S3 (annual results 2026–2040), Tables S4 and S5 (within-scenario sensitivity correlations), Table S6 (master’s-intake analysis), Table S7 (survey re-analysis), Table S8 (trainee-capacity robustness), Tables S9–S13 (further robustness and decomposition analyses) and Figures S1–S4.

### 3.3. FTE Calibration as the Methodological Foundation

The model’s methodological foundation is FTE (full-time-equivalent) logic rather than pure headcount logic. The capacity calculation uses the empirically anchored value of 945 annual treatment hours per therapist (an upper-bound reading of capacity; see Section 5.10 and Table S9) [2] and 13 treatment units per patient per year [2]. Methodologically central is the distinction between a computed FTE capacity of 72.69 persons/year per fully active therapist and an age-corrected empirical capacity of 57.31 persons/year per registered therapist. The latter—derived from the ratio 669,121/11,676—is used in the model because it implicitly accounts for the reduced activity of therapists aged over 65. This empirical calibration avoids the overestimation trap that a pure FTE approach would create.

This yields the core formulas (Appendix A.1, Table A1): annual hours = 21 h/week × 45 weeks = 945 h/year; capacity per fully active therapist = 945/13 = 72.69 persons/year; and capacity per registered therapist (model value) = 669,121/11,676 = 57.31 persons/year. Potential capacity(t) = therapists(t) × capacity per registered therapist + trainee capacity(t), where trainee capacity(t) = [i.A.u.S.(t) + PiFALS(t)] × 72.69 × [f_0_ + (f_1_ − f_0_) × insurance-funding share of trainee services × ramp(t − 1)], with f_0_ = 0.60 in the current unfunded state and f_1_ = 0.90 under full insurance funding (modes; Section 3.8.2, Appendix A.2). Accessible capacity combines the activated share of the therapists’ capacity with the insurance-funded share of the trainee capacity: Accessible capacity(t) = therapists’ capacity × activation rate(t) + trainee capacity(t) × insurance-funding share of trainee services(t). Fully funded capacity(t) = therapists’ capacity × full-funding share(t) + activated trainee capacity × (full-funding share/activation rate)—the full-funding share of therapists’ capacity is defined relative to the potential capacity throughout (cf. Code S1). In each Monte Carlo draw, the full-funding share is capped at the activation rate, so that fully funded capacity cannot exceed accessible capacity. Care gap(t) = need(t) − accessible capacity(t), which is bounded below at zero. Two distinct quantities must not be conflated at this point. The 13 treatment units per patient and year reported by Dinhof et al. [2] are the empirically observed average of currently publicly funded psychotherapeutic care in Austria; this value estimates only the capacity that the existing system realizes. The 40 sessions per year used by Riess et al. [4], by contrast, are a therapeutic reference value describing the intensity that adequate, guideline-oriented treatment of many mental disorders can require; it characterizes need but not delivered care. The model keeps the two apart: capacity is computed from the empirical 13 units, while treatment intensities of 20, 30, and 40 units are entered exclusively as sensitivity parameters in the robustness analysis (Section 4.9). This allows us to show how a higher clinical treatment intensity affects the computed capacity and care gap without altering empirical input data.

### 3.4. The Three-Level Capacity Model

The model distinguishes three operationally separate levels of care capacity. Level 1—Potential capacity: As a theoretically available care capacity based on FTE calibration, potential capacity comprises licensed professionals and, as a separate capacity line, supervised trainees of both the old (i.A.u.S.) and new systems (PiFALS, phase 3), whose treatment capacity counts as potential whether or not it is insurance-funded, though its level does rise with funding (Section 3.8.2). Level 2—Accessible capacity: The share of potential capacity translated into actually usable care via statutory-insurance contracting, outpatient-clinic structures, partial funding, or self-payment constitutes the accessible capacity; for therapists, the activation rate varies by scenario, from 33% (Scenario A) to 82% (Scenario C), and for trainees, a separate parameter—the share of trainee services funded by statutory health insurance—is being phased in from 2027 onward (Section 3.8.2). Level 3—Fully funded capacity: The share of potential capacity provided to patients without financial co-payment (statutory health insurance, ÖGK quotas, free outpatient-clinic care) constitutes the fully funded capacity; this is the socially relevant core quantity for equity-oriented care planning.

### 3.5. Chronification as a Sensitivity Parameter

The relationship between waiting times and the chronification of mental disorders is qualitatively supported by the literature, yet precise, validated risk curves by waiting duration are not available [5,6]. Two qualifications matter here: van Dijk et al. [5] found no association between waiting time and the clinical course during the waiting period—the association they did report concerns the treatment outcome—and Catarino et al. [6] modeled waiting time as a cost driver without estimating a waiting-time-dependent drop-out or persistence risk. The model therefore treats chronification as an explicit sensitivity parameter and runs three variants in parallel (Table 1); it takes the direction of the effect from the literature, and its magnitude is a scenario assumption in this study. The three variants are implemented as scenario-independent robustness runs (Section 4.9.3, Table S12): within each variant, identical chronification and waiting-time parameters apply to all three scenarios, while the main model draws these parameters from scenario-specific distributions (Table A2).

### 3.6. Monte Carlo Parameters and Distributions

All uncertain parameters are modeled as triangular distributions (minimum; mode; maximum) and drawn once per Monte Carlo iteration (Table A2). All parameters are sampled independently across iterations; conceptually related parameters (e.g., activation, trainee funding, full-funding share, and phase-3 capacity; outpatient-clinic expansion is not a separate parameter but part of the activation rate) are therefore uncorrelated within a draw, with one deliberate exception: the full-funding share is capped at the activation rate of the same draw (Section 3.3). If such parameters are, in fact, positively correlated, independent sampling understates the width of the resulting uncertainty intervals (and overstates it under a negative correlation); this simplification is acknowledged here. Throughout the study, reported intervals (95% and 80% PI, also termed uncertainty intervals in the figure captions) are percentile intervals of the simulated Monte Carlo distribution; they reflect propagated parameter uncertainties and are not repeated-sampling confidence intervals. The evidential status of each distribution—an empirically anchored value, official planning value, expert assumption, empirically informed assumption, or scenario-defining value—is classified in the notes for Table A2 and annotated per parameter in the source code (Code S1, Q1–Q5). The distributions common to all scenarios and the scenario-specific ones are listed in Table A2.

### 3.7. Scenarios

**Scenario A**—Status quo/underfunding: The current structures are continued, with a low activation rate (mean 33%), high self-payment share, and limited outpatient-clinic capacity, insurance funding of trainee services rising only to 15–50% (mode 30%; like all insurance funding of trainee services in the model, this presupposes a change in the legal or contractual framework; Section 2.3 and Section 3.8.2), and 350–800 phase-3 training places per year. **Scenario B**—Transition/partial funding: Through the stepwise expansion of outpatient structures, partial funding of trainee services (40–85%, mode 65%), 800–1600 phase-3 places per year, and the partial expansion of statutory-insurance contracting, the activation rate rises to a mean of 58% and the full-funding share to 39% of potential capacity. **Scenario C**—Expansion/full funding: With near-complete insurance funding of trainee services (75–100%, mode 90%); 1300–2500 phase-3 places at universities, outpatient clinics and teaching practices; the substantial expansion of psychotherapeutic outpatient clinics; and a marked increase in publicly funded quotas, the activation rate reaches a mean of 82% with the full-funding share at 75% of potential capacity (equivalent to 92% of accessible capacity). Master’s places (about 1000 per year: 500 publicly funded places at public universities and an estimated 500 at private universities) and admissions to the old pathway are held at their current levels in all three scenarios; the effect of additional master’s places is examined separately (Section 4.8). The empirical anchors of the FTE calibration are summarized in Table 2.

### 3.8. Workforce and Capacity Model

#### 3.8.1. Stock Dynamics

The annual change in the stock of licensed psychotherapists follows the classical stock equation: PT(t+1) = PT(t) + completions(t) − retirements(t) − exits(t). Completions come from the old pathway (until 2038) and, from 2029/2031 onward, from phase 3 of the new system. The results are reported in Section 4.5.

#### 3.8.2. Three-Level Capacity Dynamics

Potential FTE capacity results from the stock multiplied by the age-corrected capacity per registered therapist (57.31 persons/year; Section 3.3), a value that already embeds the reduced activity of therapists aged over 65 relative to the 72.69 persons/year of a fully active therapist, plus the capacity of supervised trainees. Capacity per trainee is the same for both training systems and depends on the funding for trainee services. In the current, unfunded state, it is set at 60% (range 50–70%) of a fully active therapist. This value is anchored on a stratified re-analysis of the authors’ member survey of December 2024 [9] (Supplementary Material S2, Table S7): trainees under supervision (*n* = 638) reported 0.61 of the mean weekly free-practice hours (including administration), 0.70 of the mean total weekly hours (free practice plus institution), and 0.50 of the mean annual patients of listed psychotherapists (*n* = 2139); 40% of the trainees practiced psychotherapy as their sole occupation (listed: 67%) while 82% planned to expand their hours by 2030 (mean +12.6 h/week), i.e., the unfunded trainee is a part-time provider with unused capacity. Under full insurance funding, the capacity per trainee is assumed to rise to 90% (range 80–100%)—an expert assumption of the authors, as the obligation to attend supervision remains while theory training is completed—and, because a practice has to be built up, it follows the funding ramp with a lag of one year, expressed as capacity per trainee(t) = 72.69 × [f_0_ + (f_1_ − f_0_) × funding share × ramp(t − 1)] and applied to the entire treating trainee stock. With the scenario-specific funding shares, the effective factor therefore rises from 0.60 in 2026–2027 to about 0.70 (A), 0.79 (B), and 0.86 (C) by 2031 (medians); both f_0_ and f_1_ are part of the sensitivity analysis, and fixed funding-independent factors are examined in Section 4.9.2. Accessible capacity is the sum of therapists’ capacity multiplied by the scenario-dependent activation rate and trainee capacity multiplied by the share of trainee services funded by statutory health insurance. Trainee services are currently paid privately—under the current law, they are not an insurance benefit (Section 2.3)—so their insurance funding presupposes a change in the legal or contractual framework; the model therefore treats it as a policy measure that can take effect after the register listing of 1 October 2026, and the funding share is phased in linearly from zero in 2026 to its scenario value by 2030 (25% of the target in 2027, 50% in 2028, 75% in 2029). In Scenario A, the activation rate is only 33% (corresponding to today’s reality of roughly 30% of all therapists holding an insurance quota or an outpatient-clinic position). In Scenario C, it rises to 82%, implying a comprehensive restructuring of the care landscape, with substantial insurance expansion and outpatient-clinic development.

#### 3.8.3. Care Gap

The care gap is operationalized as the positive difference between need (treatment willingness × population) and accessible capacity: care gap(t) = max(0, need(t) − accessible capacity(t)).

### 3.9. Reform-Cohort Model

The reform-cohort model distinguishes three groups simulated in parallel (Table 3): (1) persons in the old training system (Fachspezifikum), anchored on the training statistics from 2025 (5601 enrolled, 3238 of them i.A.u.S.) [14], with admissions of about 950 per year until the statutory admission deadline of 1 October 2030, completions following the observed turnover of the pool (about nine years) until 2030 and accelerating thereafter, such that all remaining candidates complete by the statutory deadline of 30 September 2038 (§§ 60(4) and 61(3) PThG 2024)—the i.A.u.S. share of the pool is 57.8% until 2030 and rises to 100% by 2033 as the remaining candidates advance into the supervised-treatment phase; (2) master’s-entry cohorts of the new system from the winter semester 2026/27 onward—about 1000 per year (500 fixed places at public universities, 250–750 at private universities), with two years of master’s studies, followed by phase 3 (PiFALS) for a duration of two to five years and a lead time of 0.5–2 years before full treatment activity—supplemented by a small number of direct entrants to phase 3 under § 10 PThG 2024 (20–100 per year), with the completion rate of the new system (Table A2) applied at the end of the master’s program; and (3) bachelor’s programs of the new system, currently offered only at private universities, whose graduates fill the same master’s places and are therefore not counted separately. Propaedeutic candidates who can no longer enter the old Fachspezifikum in time (about 30% switch to the master’s program, according to the authors’ assessment) compete for the existing master’s places and do not add throughput. Phase-3 training places (Table A2, places per year) multiplied by the phase-3 duration produces the number of simultaneously available places; candidates without a place wait in a first-in-first-out queue and are treated at 50% of the PiFALS capacity while waiting (500 of the 1000 required treatment hours can be completed outside a formal training place). This waiting time prolongs their training. The queue logic is adapted from the authors’ phase-3 decision model; all candidates who begin phase 3 complete it.

This structure implies two transition phenomena. The first is the familiar ‘reform dip’ in licensures between the end of the old pathway and the ramp-up of the new one; the second is a ‘transition cliff’ in 2038, when the old pathway’s completions and supervised trainees disappear within one to two years. Both are quantified in Section 4.5 and discussed in Section 5.3.

### 3.10. Health-Economic Model

Societal follow-on costs comprise three additive components: follow-on costs(t) = waiting-time costs(t) + chronification costs(t) + productivity losses(t). This additive structure does not fully capture the interaction effects between components (e.g., chronification increasing sick leave).

**Waiting-time costs** cover the excess utilization of general practice, psychiatric emergency care, and psychotropic medication between care indication and treatment start. Based on the international evidence regarding excess costs and productivity effects of untreated and more severe mental disorders [7,8], the base costs of €2500/€5000/€9000 per untreated person and year are modeled (triangular; mode €5000) and multiplied by the scenario-specific waiting-time cost factor (Table A2), which corresponds to effective waiting-time costs of about €440–€710 per untreated person and month across the scenarios. **Chronification costs** arise because chronic mental disorders entail substantially higher lifetime costs; the model computes them as the product of the care gap, the chronification rate (variant-dependent), and an excess cost factor (triangular €4000/€10,000/€20,000 per chronified person and year, mean about €11,300; Table A2). To orientate this, König et al. [8] reported six-month excess costs of €6123 (severity level 1) to €31,883 (level 4) in 2019 euros, i.e., roughly €12,000 to €64,000 annualized; the mode and mean used here are deliberately set below the lower end of that range. **Productivity losses** follow the human-capital approach, where an employment rate of 58%, 24 sick-leave days/year, and a GDP contribution of €490 per working day imply about €6800 per affected person and year (24 × €490 ≈ €11,800 per affected employed person, weighted by the employment rate). Because only some people in the care gap incur losses of that magnitude, this value enters the model as an upper anchor. In contrast, the modeled productivity cost per person in the care gap is drawn from a triangular distribution of €1000/€3000/€7000 (Table A2). For context, in 2025, dependent employees in Austria recorded an average of 14.7 absence days per insured person, corresponding to about 4.0% of total working time [15]; the 24 days assumed here refer to persons with untreated mental disorders, whose absence durations exceed the population’s average.

### 3.11. Empirical Anchoring of Secondary Costs: Austrian Cost Data

To anchor the per-person cost parameters in Austrian empirical data, we cross-checked the model’s cost assumptions against the secondary-cost compilation by Seitz et al. [16], who assembled per-patient primary and secondary annual costs for chronic pain patients with somatic symptom disorder in Austria (*n* = 106; 1-year follow-up after an 8-week integrated inpatient intervention) from Austrian statistical and tariff sources: €186 per sick-leave day, derived from an average of 35 million workdays lost per year at a total cost of €6.5 billion over 2006–2010 [16,17]; €3168 per hospital day in a public hospital (range: €2923–3401); €24 per medical examination and €10.1 per physician consultation billed to the social-insurance carriers; and average medication costs per patient [16].

In that high-utilizer population, the total annual secondary costs per patient amounted to €147,689 before the intervention and €67,943 in the year after it—a reduction of €79,746 per patient per year, driven mainly by 24.3 fewer hospitalization days (−€76,919) and 64.5 fewer sick-leave days (−€11,980), although the expenditure on psychotherapy sessions increased (+€237) [16]. Table 4 sets these empirical anchors against the model’s per-person cost parameters.

## 4. Results

### 4.1. Scenario Analysis

**Scenario A**—Status quo/underfunding continues current structures. The activation rate remains at about 33%; only about 15% of potential capacity is provided fully funded. The care gap falls from about 418,000 persons/year in 2026 to about 287,000 in 2033—even the modest insurance funding of trainee services (30%) activates up to 84,000 persons/year of trainee capacity—before widening again to about 321,000 by 2040 as the old-system trainees complete and disappear (§ 60 PThG 2024). The new system delivers only about 580 licensures per year. Cumulative follow-on costs by 2040: €83 billion (95% PI: 37–143).

**Scenario B**—Transition/partial funding assumes a stepwise expansion of outpatient structures and partial funding of trainee services (65%). The activation rate rises to 58%, the full-funding share to 39% of potential capacity. The care gap falls from 249,000 (2026) to zero between 2032 and 2036—activated trainee capacity peaks at about 197,000 persons/year in 2032—and reopens to about 37,000 persons/year in 2040 after the old pathway ends (38% of runs show no gap in 2040); relative to Scenario A, this is an 88% reduction. Cumulative follow-on costs: €13 billion (95% PI: 1–51).

**Scenario C**—Expansion/full funding models the near-complete insurance funding of trainee services (90%), sufficient phase-3 training places, and a substantial expansion of psychotherapeutic outpatient clinics. The activation rate reaches about 82%, with the full-funding share at 75% of potential capacity (equivalent to 92% of accessible capacity). Accessible capacity exceeds the median modeled need from 2028; the care gap remains at a median of zero until 2040 (99% of runs show no gap in 2040). Cumulative follow-on costs fall to €1.3 billion (95% PI: 0–8), corresponding to avoided follow-on costs of roughly €82 billion versus Scenario A.

Timing these levers is decisive. Insurance funding of trainee services acts within one to four years, as trainees already exist and are already treating; the phase-3 and master’s pipeline acts on licensures after four to seven years; and the statutory end of the old pathway in 2038 removes both its completions and its trainees regardless of policy. Measures that expand phase-3 places and master’s places would therefore need to be in place well before 2038 if the workforce is to keep growing after the transition (Section 4.8).

### 4.2. Main Results of the Monte Carlo Simulation

Table 5 summarizes the main results of the FTE-calibrated Monte Carlo simulation for the final year of 2040.

**Table 5:** Main results of the FTE-calibrated Monte Carlo simulation (final year 2040).

Indicator (Median, 2040)	Scenario A (Status quo/Underfunding)	Scenario B (Transition/Partial Funding)	Scenario C (Expansion/Full Funding)
Cumulative follow-on costs (€ million)	83,437	13,136	1339
95% PI, follow-on costs (€ million)	37,095–143,252	1433–50,720	0–8123
Care gap (persons/year)	320,736	36,951	0
95% PI, care gap (persons/year)	142,273–475,350	0–247,225	0–0
Potential capacity (persons/year)	980,339	1,047,308	1,104,651
Accessible capacity (persons/year)	320,417	603,274	907,552
Fully funded capacity (persons/year)	151,919	412,361	831,359
Activation rate (accessible/potential)	33%	58%	82%
Licensed psychotherapists	15,333	16,743	17,439
Supervised trainees, old system (i.A.u.S.; peak 2032)	0 (4214)	0 (4214)	0 (4214)
Phase-3 trainees, new system, in a training place (PiFALS)	1788	2431	2739
Phase-3 candidates waiting for a training place	1046	0	0
Trainee capacity, potential (persons/year; peak 2032/33)	88,224 (268,415)	68,523 (310,708)	84,249 (349,358)
Trainee capacity, insurance-funded (persons/year; peak)	26,352 (84,338)	42,384 (197,135)	74,210 (308,193)
Capacity factor per supervised trainee (share of a fully active therapist; 2026 → 2040)	0.60 → 0.70	0.60 → 0.79	0.60 → 0.86
Completions, old system	0	0	0
Completions, new system	576	744	838
Retirements/exits	832	902	937
Mean waiting time for a phase-3 place (years, admitted candidates)	0.7	0.0	0.0

Note: All cost values in € million (2024 price basis), cumulative 2026–2040. PI = prediction interval. Activation rate = accessible capacity/potential capacity (ratio of the medians shown; the modes of the scenario-specific activation parameter for therapists are 32%/58%/82%, Table A2). The full-funding share is defined relative to potential capacity (A 15%, B 39%, C 75%). Potential capacity includes the full treatment capacity of supervised trainees. The old-system trainee stock (i.A.u.S.) is zero by 2040 in all scenarios because the statutory completion deadline (30 September 2038) has passed; its peak (2032) is given in parentheses. Phase-3 trainees in Scenario A are limited by the training places (350–800 per year, Table A2). Data source: MC_FTE_Kalibriert_Summary_2040 (*n* = 30,000 iterations).

### 4.3. Capacity and Need in the Final Year of 2040

**Figure 1:**
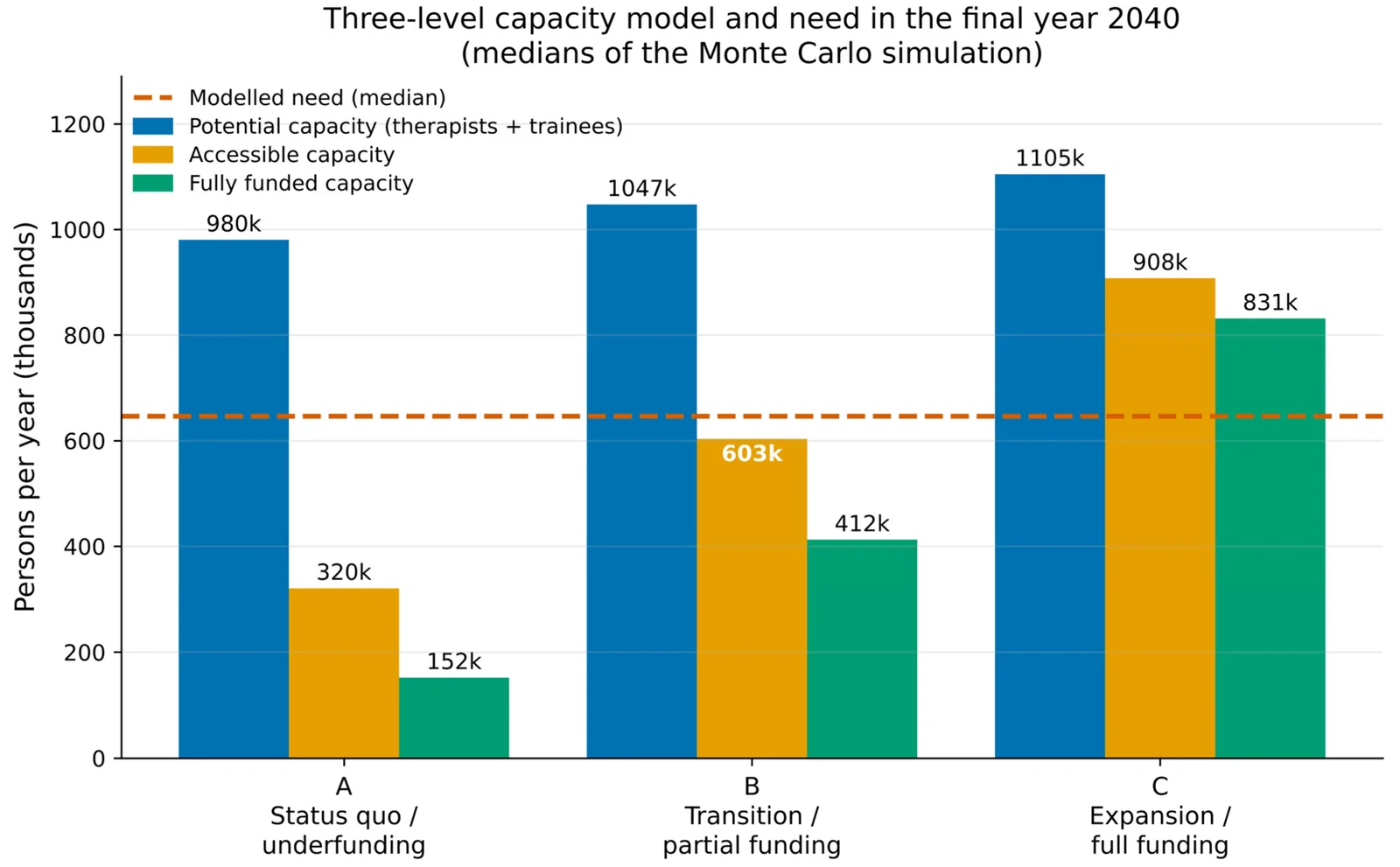
Three-level capacity model and need in the final year of 2040 by scenario (medians of the Monte Carlo simulation); potential capacity includes the treatment capacity of supervised trainees. Source: Authors’ FTE-calibrated Monte Carlo simulation.

Figure 1 presents the central three-level capacity model in the final year of 2040.

The figure visualizes the central structural finding of this study: the potential capacity exceeds need in every scenario in 2040 (Scenario A: 980,000 persons/year against a median need of about 647,000, i.e., 152%) because the licensed profession grows until 2038 and because the treatment capacity of supervised trainees counts as potential; yet in Scenario A, the accessible capacity (320,000) covers only 50% of need and fully funded capacity (152,000) covers only 23%. In Scenario B, the accessible capacity (603,000) reaches 93% of need, and fully funded capacity (412,000) reaches 64%. In Scenario C, accessible capacity (908,000) exceeds need (140%) and fully funded capacity (831,000) alone covers 129%.

This illustrates why a pure workforce policy remains insufficient: with the same professions and the same trainees, the three scenarios differ in 2040 by a factor of almost three in terms of accessible care. The difference between potential and accessible capacity is the policy-relevant activation margin, and in 2040, between 6.5% and 9% of potential capacity (88,000/69,000/84,000 persons/year in A/B/C) is the trainee capacity that depends on a single lever—the insurance funding of trainee services, which, in the model, raises the activated share and the capacity per trainee.

### 4.4. Cumulative Societal Follow-On Costs

**Figure 2:**
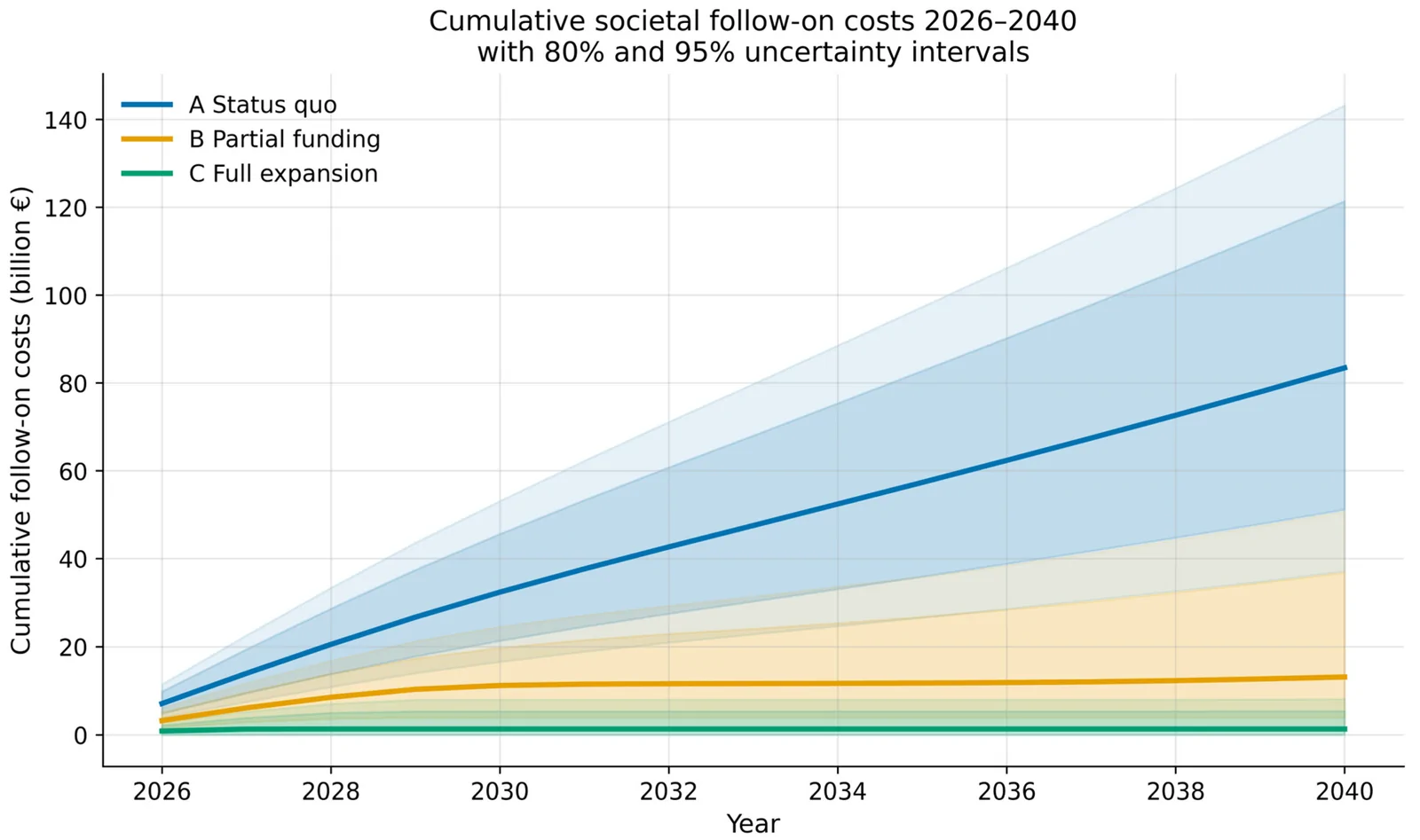
Cumulative societal follow-on costs during 2026–2040, with 80% and 95% uncertainty intervals, calibrated to the FTE model. Lines show medians; darker shaded bands show the 80% intervals (10th–90th percentiles) and lighter bands the 95% intervals (2.5th–97.5th percentiles) in the colour of each scenario. Source: Authors’ Monte Carlo simulation (*n* = 30,000).

Figure 2 shows the cumulative follow-on costs from 2026 to 2040, with 80% and 95% uncertainty intervals per scenario.

The follow-on cost curves diverge strongly with the increasing projection horizon (medians and intervals in Section 4.1).

It is worth noting the saturation dynamic in Scenarios B and C: because accessible capacity covers the median needs from 2028 (C) and 2032 (B), annual follow-on costs fall to zero from these points, and the cumulative cost curve flattens into a plateau; in Scenario B, the curve resumes a slight rise from 2037, which is when the old-system trainees have completed, and the gap reopens.

Even when accounting for the uncertainty intervals, the scenario ranking remains robust: the upper 95% bound of Scenario C (€8 billion) lies clearly below the lower 95% bound of Scenario A (€37 billion), and Scenario C is cheaper than Scenario A in 100% of simulation runs and cheaper than Scenario B in 94% of them (Scenario B cheaper than A in 99%; unpaired comparison of runs across scenarios). In the model, activation and full funding are associated with substantially lower societal follow-on costs than maintaining the status quo under every plausible parameter constellation.

### 4.5. Reform-Cohort Dynamics: Completions and Retirements

**Figure 3:**
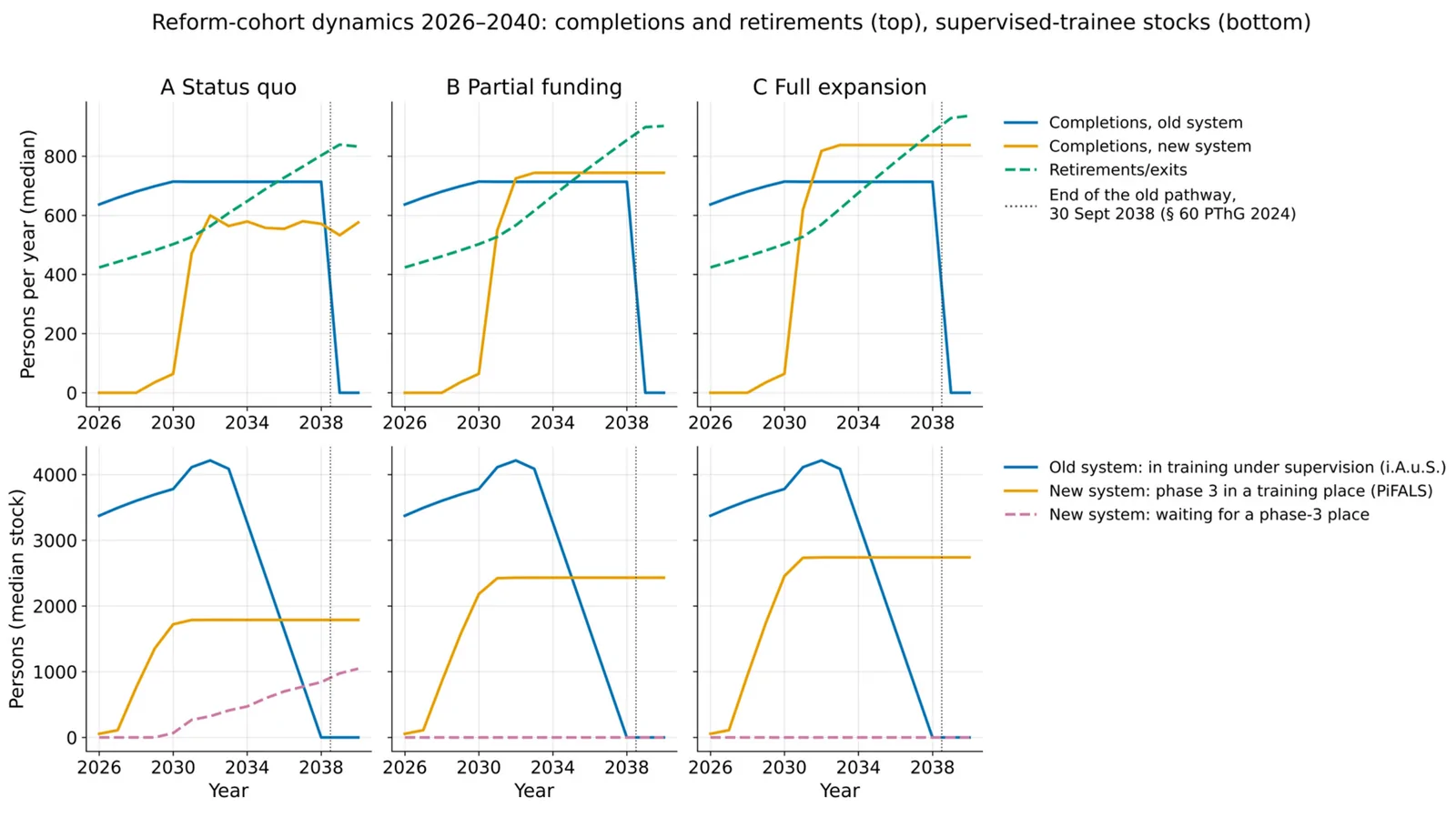
Reform-cohort dynamics 2026–2040 by scenario (medians): completions of the old and new systems and retirements/exits (top row); stocks of supervised trainees in the old system, in phase-3 training places, and waiting for a place (bottom row). Dotted line: End of the old pathway (30 September 2038). Source: Authors’ simulation.

Figure 3 visualizes the interplay of completions from the old system, completions from the new system, and retirements/exits (top row), as well as the stocks of supervised trainees in the old system, in phase-3 training places, and in the waiting queue (bottom row), across the three scenarios.

The graph shows that the old pathway, fed by about 950 admissions per year until 2030, delivers about 640 completions in 2026 followed by about 714 per year from 2030 to 2038—slightly above the 624 observed in 2024/25—before it ends under the transitional law (§ 60 PThG 2024). Completions from the new system begin with a few direct entrants in 2029, reach about 470–620 in 2031, and plateau at about 580/year in A, 740/year in B and 840/year in C from 2033; the differences between scenarios stem from the completion rate and, in Scenario A, from the phase-3 places, which become binding from 2030—about 1050 candidates are waiting for a place in 2040 (mean waiting time 0.7 years), whereas in B and C, the places suffice. The bottom row shows the trainee stocks: the old-system stock rises from about 3400 (2026) to a peak of about 4200 in 2032 and falls to zero by 2039, while the new-system phase-3 stock builds up from 2028 to about 1800/2400/2700 trainees (A/B/C) by 2031–2032—a level limited in A by places and in B and C by master’s intake.

The retirement line rises in all scenarios from about 420 (2026) to 830–940 per year (2040) as the stock grows; until 2038, completions exceed retirements everywhere, such that the licensed stock grows to 16,200–17,900 by 2038. From 2039, the picture reverses: without the old pathway, the new system’s 580–840 licensures per year fall short of retirements, and the stock declines by roughly 240–430 per year (C/A). The ‘retirement wave’ is therefore not the binding constraint of the transition decade; the binding constraint is the new system’s throughput after 2038.

### 4.6. Care Gap with Uncertainty Intervals

**Figure 4:**
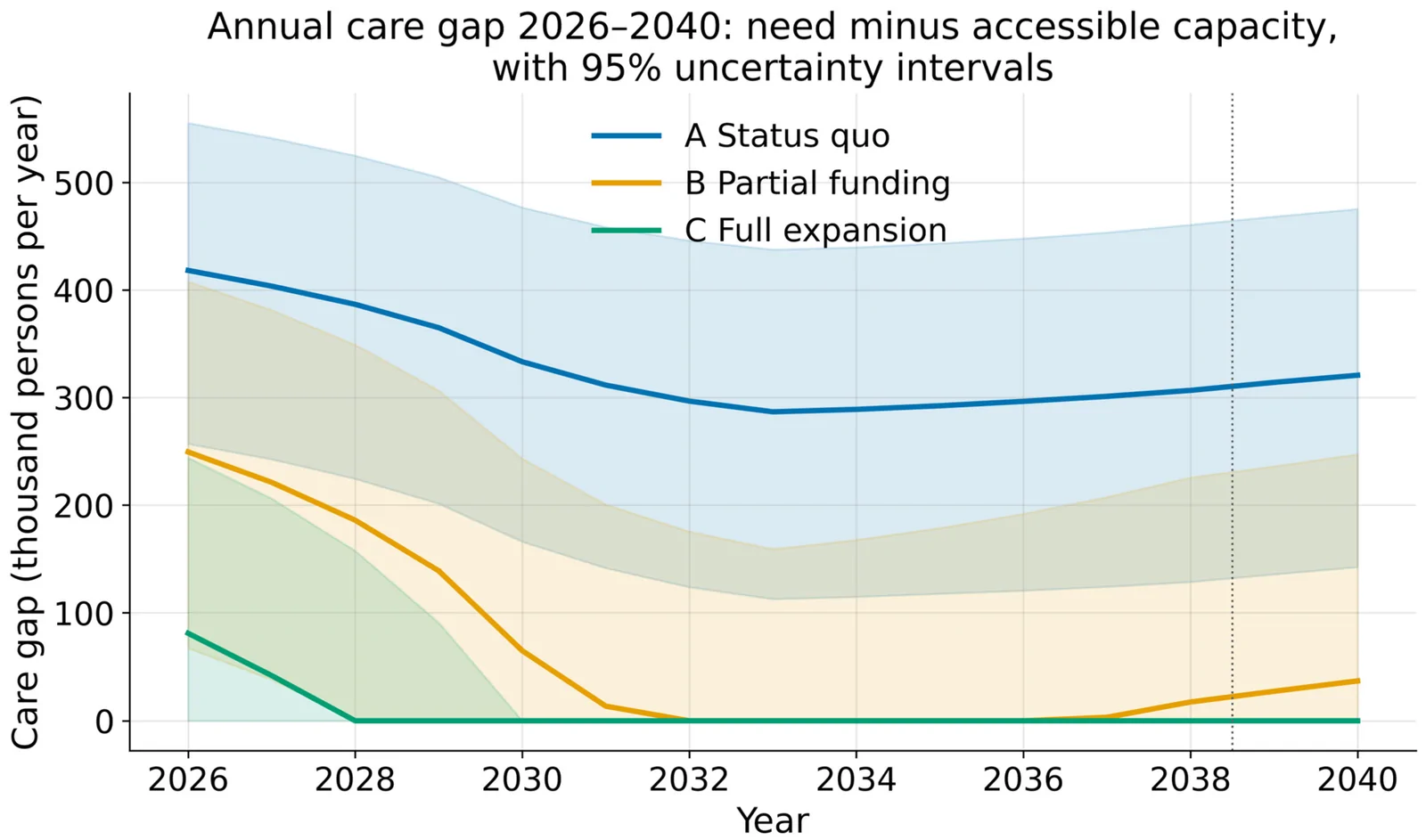
Annual care gap 2026–2040: need minus accessible capacity, with 95% uncertainty intervals. Lines show medians; shaded areas show the 95% uncertainty intervals (2.5th–97.5th percentiles) in the colour of each scenario (colours blend where intervals overlap). Dotted line: end of the old pathway (30 September 2038). Source: Authors’ simulation.

Figure 4 shows the development of the annual care gap—operationalized as need minus accessible capacity—over 2026–2040, with 95% uncertainty intervals per scenario.

The three scenario curves show marked qualitative differences (medians in Section 4.1); in 2040, 99% of Scenario C runs and 38% of Scenario B runs show no gap.

The most important clinical–structural result is the speed at which trainee funding acts: both B and C close the median gap within six years, without a single additional licensure, because they activate capacity that already exists. The wide uncertainty bands reflect the dependence of this result on the level of needs, the activation rate, and the assumed trainee capacity; however, they are tellingly asymmetric in favor of covering need, which is bounded below at zero.

### 4.7. Sensitivity Analysis

**Figure 5:**
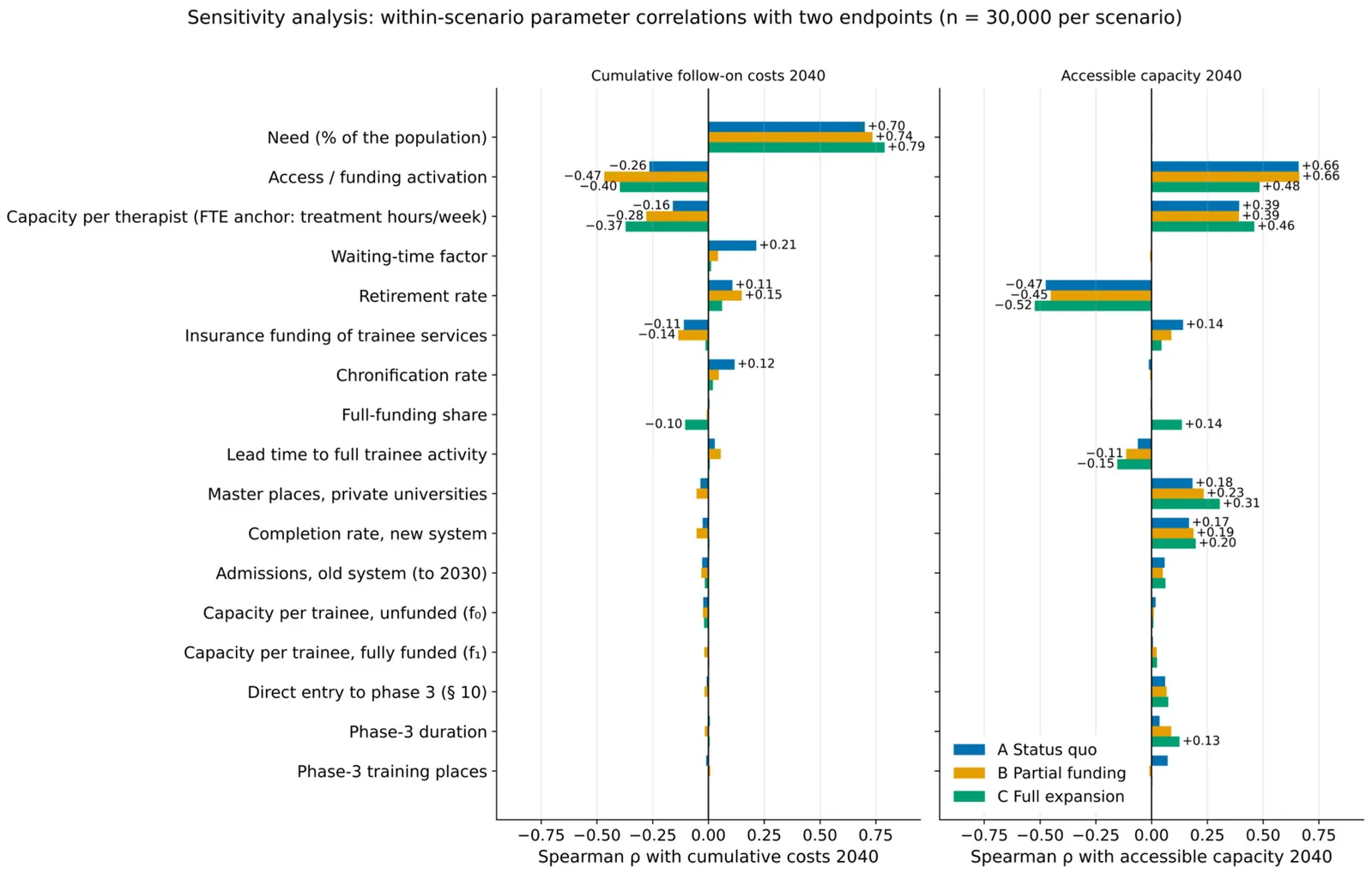
Sensitivity analysis: Within-scenario Spearman rank correlations of the 17 model parameters with cumulative follow-on costs by 2040 (left) and with accessible capacity in 2040 (right); n = 30,000 per scenario. Source: Authors’ simulation.

Figure 5 quantifies the influence of the 17 uncertain model parameters on two endpoints—cumulative follow-on costs by 2040 and accessible capacity in 2040. In the figure, Spearman rank correlations computed within each scenario across its 30,000 Monte Carlo iterations are shown: positive values indicate increasing effects of higher parameter values, while negative values indicate decreasing effects (Table 6). We compute correlations within scenarios because the scenario-specific parameter distributions barely overlap; a correlation pooled across scenarios would measure scenario membership rather than parameter influence and is therefore not reported. We report two endpoints because the training-pipeline parameters affect the workforce and capacity with a lag and are therefore invisible in the cost endpoint, which is dominated by the years before 2032. The weekly-hours anchor enters this analysis through the capacity per therapist (FTE anchor): its distribution (50–68 persons/year per registered therapist, mode 57.31; Table A2) corresponds to scaling the 21-h anchor by a factor of 0.87–1.19, i.e., to about 18–25 treatment hours/week per therapist at 13 units per patient per year. The alternative anchors of 15.8 and 13.3 treatment hours/week lie outside this range and reflect a different measure (activity-adjusted means instead of the published median); they are therefore analyzed as separate model runs rather than as sampled uncertainty (Section 4.9.3, Table S9).

The sensitivity analysis yields five central insights. First, the choice of policy package dominates: cumulative costs differ between the scenarios by more than an order of magnitude, a between-scenario finding carried by the scenario comparison (Section 4.1 and Section 4.4). Second, within every scenario, the assumed population need is the largest single source of cost uncertainty (ρ = +0.70/+0.74/+0.79), and its weight increases with the activation level; closing the gap does not buffer the system against need as it makes need the binding uncertainty, which is why the robustness analysis across need levels (Section 4.9) is integral to the model rather than an appendix to it. Third, regarding costs, the activation rate is the strongest steerable parameter, followed by the FTE anchor, the retirement rate, and the insurance funding of trainee services; the clinical risk factors (waiting-time factor, chronification) matter only in the underfunded Scenario A (Table 6). Fourth, for accessible capacity in 2040, the ranking changes: after the activation rate, the retirement rate, and the FTE anchor, the pipeline parameters become visible (master’s places, completion rate, insurance funding of trainee services, lead time to full trainee activity, phase-3 duration; Table 6); for the licensed stock in 2040, the retirement rate dominates (ρ ≈ −0.8), followed by master’s places, the completion rate, and, in Scenario A alone, by the phase-3 places, which bind there (Table S5). Fifth, the full-funding share does not influence costs or the care gap by construction (its non-zero correlations in Scenario C, −0.10 with costs and +0.14 with accessible capacity, arise only from its coupling to the activation rate through the consistency constraint): it defines the equity level of care (level 3) and must be read as a distributional, not an efficiency, parameter; likewise, the two trainee-capacity parameters—the unfunded level f_0_ (0.5–0.7 of a fully active therapist) and the fully funded level f_1_ (0.8–1.0)—have only small within-scenario correlations with costs and accessible capacity (|ρ| ≤ 0.03), even though they set the level of the trainee effect, as their uncertainty ranges are narrow relative to the need range. Their influence lies in the level of the results, not in their dispersion, which is why the trainee-capacity assumption is examined separately with fixed factors in Section 4.9.2.

### 4.8. How Many Master’s Places Does the New System Need?

The new system’s master’s intake is currently limited to about 1000 places per year (about 500 publicly funded places at public universities and an estimated 500 at private universities; Section 3.9). To determine the intake at which the new system sustains the workforce after the old pathway ends, the master’s intake was varied from 1000 to 2500 places per year in all three scenarios, with all other draws unchanged (Table 7). Because phase-3 places are the second bottleneck, we ran each intake level twice: with the phase-3 places in the respective scenarios (Table A2) and with phase-3 places scaled in proportion to the intake.

Three results follow. First, with proportionally growing phase-3 places, Scenario B closes the care gap durably with about 1500 master’s places per year, and Scenario C from the current intake; Scenario A—with today’s activation and trainee funding—does not close it at any intake examined (168,000 persons/year at 2500 places). Second, master’s places alone are not enough: with the phase-3 places of Scenario A held constant, an intake of 2500 produces a waiting queue of about 12,000 candidates in 2040 (mean waiting time 3.5 years), with a smaller licensed stock and a larger gap than the same intake with proportional phase-3 places (Table 7 and its notes). Third, with every additional 500 master’s places, the licensed stock in 2040 is raised by about 2000–3000 persons (A/B/C), i.e., the throughput needed to replace the old pathway’s 714 completions per year and to keep the stock growing after 2038 lies at roughly 1500 places per year, provided that the phase-3 places grow with it.

### 4.9. Robustness Analysis: Need, Treatment Intensity and Trainee Capacity

The results in Section 4.1, Section 4.2, Section 4.3, Section 4.4, Section 4.5, Section 4.6, Section 4.7 and Section 4.8 rest on the assumption of a population need of up to 9% (range 5–9%, mode 7.5%) and a treatment intensity of 13 units per patient per year, with the latter matching the value on which the capacity anchor of Dinhof et al. is based [2]. Both assumptions are open to challenge: published Austrian estimates span a clinical need of about 13.8% of the population and a realistic treatment need of about 9% [4]—the four need levels examined here (5%, 7.5%, 9%, 12%) are therefore an explicit modeling choice spanning a deliberately low lower bound and an upper bound that includes subclinical burden and not a range taken from a single source—while Riess et al. [4] used 40 sessions per year as a therapeutic reference value for guideline-oriented treatment; because adequate intensity varies with diagnosis, severity, treatment modality and treatment goals, 30 and 40 units per year are treated here as higher-intensity scenarios and not as a general benchmark.

To test the stability of the model results against these two central parameter assumptions, a systematic robustness matrix was computed—four need levels (5%; 7.5%; 9%; 12%) × four treatment intensities (13; 20; 30; 40 units) × three scenarios = 48 constellations—with each being deterministic and with additional Monte Carlo uncertainty (20,000 iterations) around the activation and funding parameters.

The capacity scaling follows a simple, transparent logic. Since theoretical total capacity (669,121 persons/year) is based on 13 units per patient per year, it scales inversely proportionally to treatment intensity: capacity(E) = 669,121 × (13/E). At E = 20 units, theoretical capacity falls to 435,000 persons/year; at E = 30, to 290,000; and at E = 40, to ~217,000. This deterministic reduction is mathematically necessary and cannot be compensated by activation. The matrix is a static analysis: it rescales the 2023 capacity anchor (669,121 persons/year: the theoretical capacity of the 11,676 licensed psychotherapists of 2023 at 13 units/patient/year, without trainee capacity) and applies fixed scenario activation shares to it (30%/57%/82% for A/B/C in the deterministic matrix, varied as triangular distributions in the Monte Carlo component; Supplementary Material S3, Section S3.8.4) instead of using the dynamically projected workforce and trainee stocks of Section 4.1, Section 4.2, Section 4.3, Section 4.4, Section 4.5, Section 4.6, Section 4.7 and Section 4.8; the same intensities applied to the projected capacities of 2030 and 2040 are reported in Section 4.9.3 (Table S10).

#### 4.9.1. Coverage by Need Level and Treatment Intensity (Static Robustness Matrix)

Figures S1–S3 show the percentage coverage (accessible capacity/need) for each scenario across all 16 need-by-intensity constellations. Values ≥ 100% indicate full need coverage; values clearly below indicate a substantial care gap.

In Scenario A, coverage reaches at most 45% of need (5% need, 13 units).

In Scenario B, near-complete coverage (85%) is reached only at 5% need and 13 units.

Scenario C is the only one in which coverage exceeds 100% (123% at 5% need and 13 units); at 40 units and 12% need, it remains at 17%.

**Figure 6:**
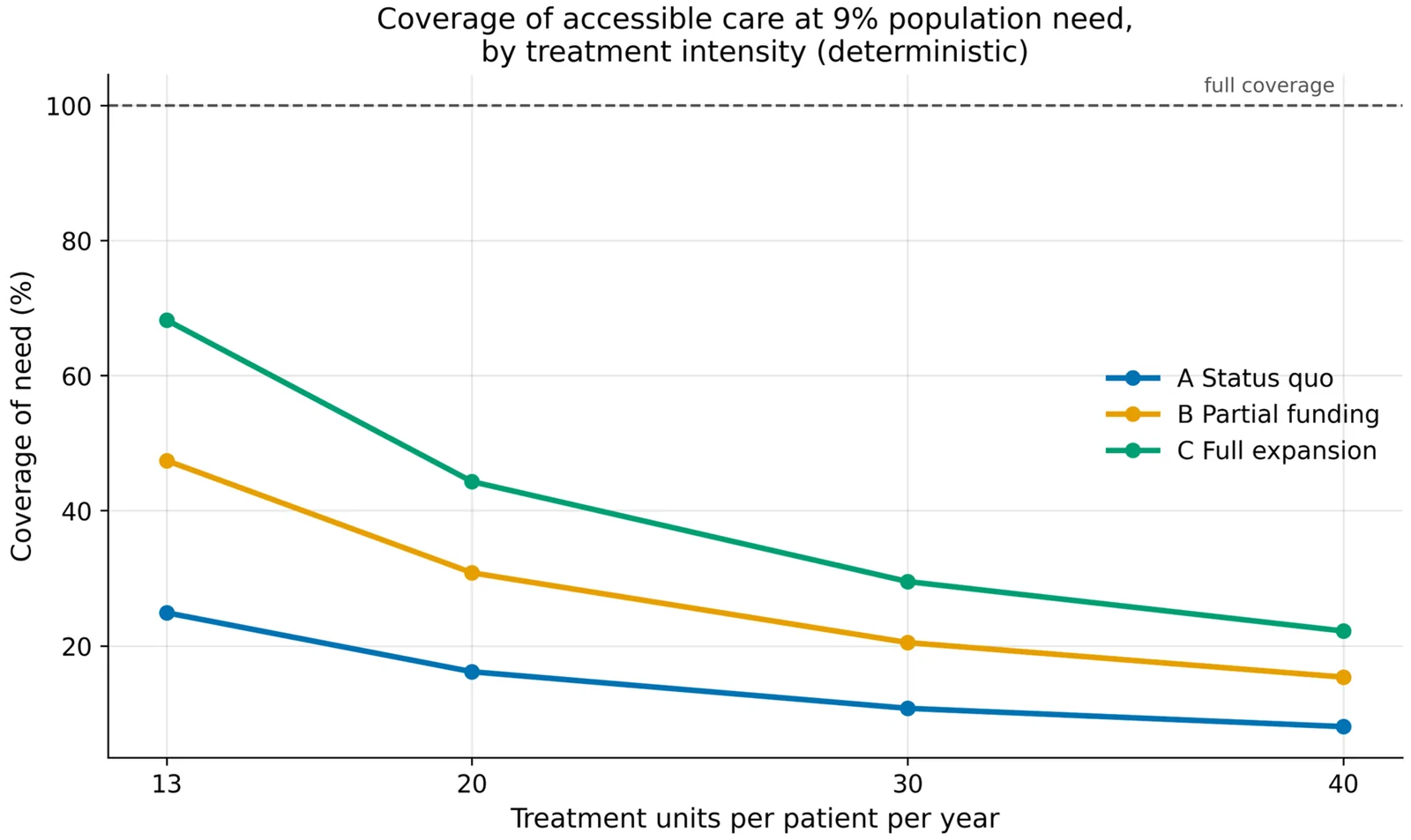
Coverage of accessible care at 9% need as a function of treatment intensity (deterministic calculation at the static 2023 capacity anchor of 669,121 persons/year; for the dynamically projected capacities, see Table S10). Source: Authors’ simulation.

Figure 6 focuses on the policy-central need level of 9% and shows coverage as a function of treatment intensity.

The graph makes the central robustness finding tangible: all three scenario curves fall markedly as treatment intensity rises. While Scenario C still achieves 68% coverage at 13 units, this value falls to 30% at 30 units and 22% at 40 units. The scenario difference (C–A) persists in absolute terms but shrinks relatively; at high treatment intensity, activation alone becomes an insufficient lever.

The absolute gap (Figure S4) grows monotonically with treatment intensity: in Scenario C, it rises from about 256,000 persons (13 units) to about 567,000 (30 units) and to about 627,000 (40 units). In absolute numbers, this gap is comparable to that of Scenario A at low intensity, highlighting treatment intensity as an underestimated model parameter.

**Figure 7:**
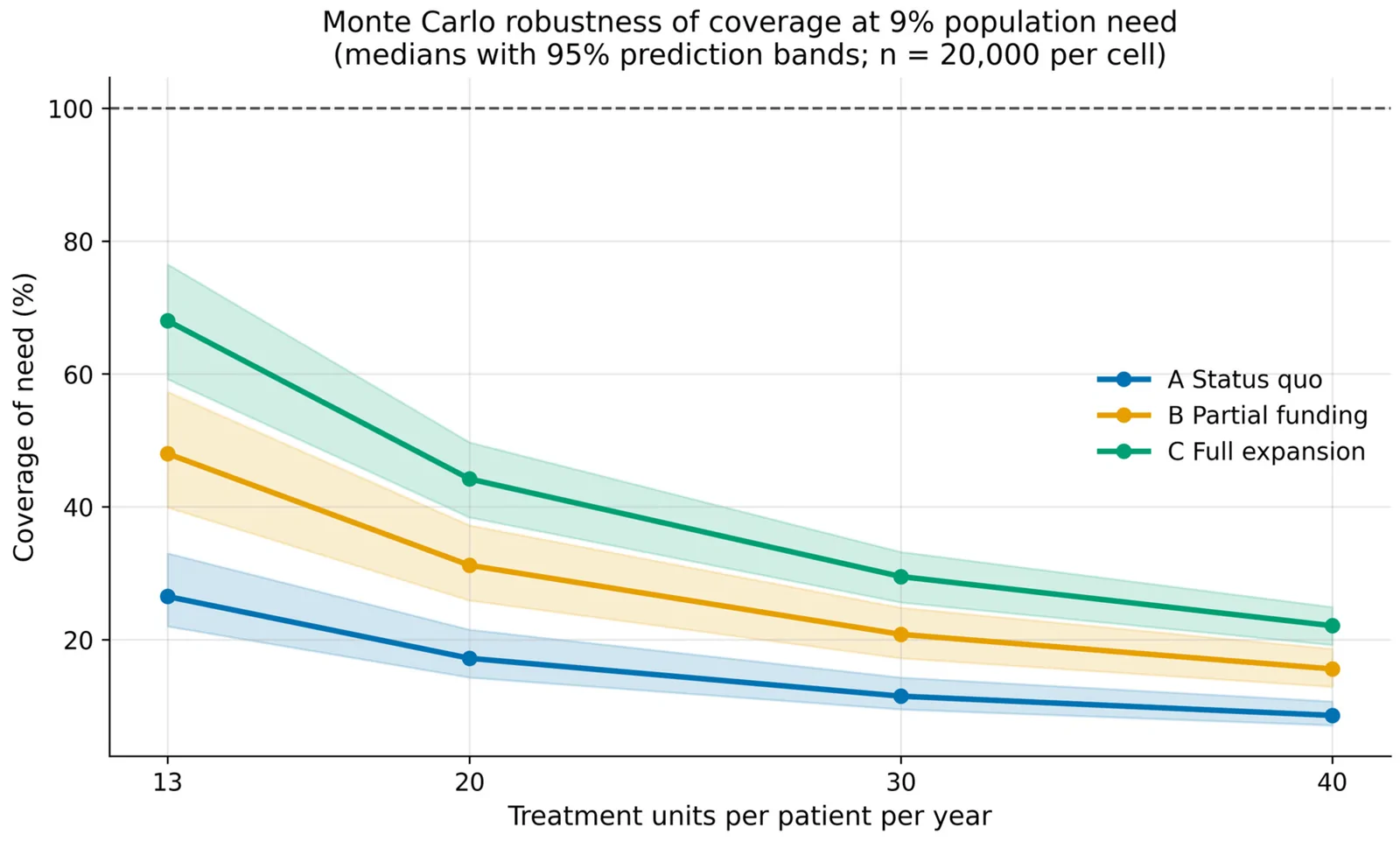
Monte Carlo robustness of coverage at 9% need by treatment intensity at the static 2023 capacity anchor, with 95% uncertainty intervals (derived from propagated parameter uncertainty, not repeated-sampling confidence intervals). Lines and markers show medians; shaded bands show the 95% uncertainty intervals. Source: Authors’ simulation (*n* = 20,000 per cell).

The deterministic robustness matrix was complemented by a Monte Carlo component in which the scenario-specific activation and full-funding shares vary as triangular distributions (20,000 iterations per cell). Figure 7 presents the resulting medians and 95% uncertainty intervals for coverage at 9% need.

The uncertainty intervals clarify three findings: (1) the scenario ranking is robust under all treatment intensities—the 95% uncertainty intervals do not overlap; (2) with rising treatment intensity, the absolute intervals narrow, reflecting the approach to a structural care shortage that parameter fluctuations can barely alter; and (3) even the upper bound of the 95% uncertainty interval of Scenario C at 30 units does not reach the 100% line.

**Figure 8:**
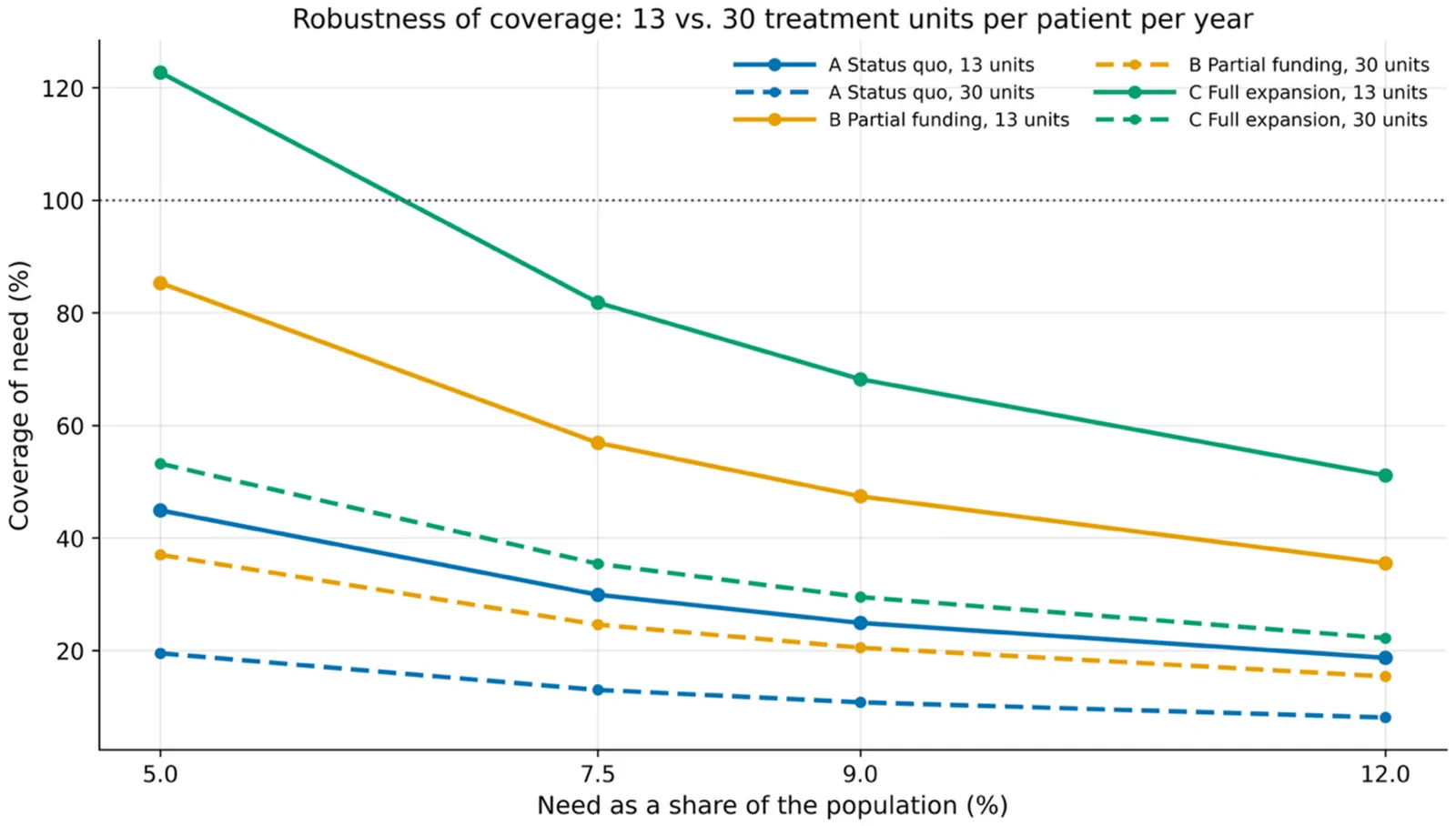
Direct comparison of coverage at 13 vs. 30 units per patient per year across need levels of 5–12% (static 2023 capacity anchor). Source: Authors’ simulation.

Figure 8 presents coverage at two contrasting treatment intensities (13 units = current value; 30 units = higher-intensity scenario) across all need levels. Because capacity is proportional to the weekly treatment hours divided by the units per patient per year (capacity = 669,121 × (h/21) × (13/E)), a lower weekly-hours anchor acts like a higher treatment intensity: at 13 units per patient per year, the alternative anchors of 15.8 and 13.3 treatment hours/week are equivalent to about 17 and 21 units at the 21-h anchor (Scenario C, 9% need: about 51% and 43% coverage instead of 68%; for the dynamically projected model, see Section 4.9.3 and Table S9).

The graph shows the model’s most important methodological asymmetry: at 13 units, Scenario C can computationally cover even higher need levels (123% at 5% need; 51% at 12% need). At 30 units, all three scenario curves remain permanently below the 60% line. The difference between the two treatment intensities is larger in every scenario than the difference between the scenarios themselves—a central finding for interpretation.

#### 4.9.2. Robustness to the Trainee-Capacity Assumption

The capacity per supervised trainee is the least documented quantity of the model (Section 3.8.2). To test how far the results depend on it, the main model—in which the capacity factor rises with the insurance funding of trainee services from f_0_ ≈ 0.60 to at most f_1_ ≈ 0.90—was compared with four variants in which the factor is fixed at 0.3, 0.5, 0.7, or 0.9 of a fully active therapist, for both training systems and irrespective of funding, and with a counterfactual lower bound in which trainee services are never insurance-funded in any scenario (funding share zero; capacity per trainee stays at f_0_; this corresponds to the current legal situation, Section 2.3), with all other draws unchanged (Table 8). The fixed factors bracket the survey-based unfunded level (0.50–0.70) from below (0.3) and reach the mode of the fully funded level (0.9), which lies above the effective factor of every scenario in the main model (at most 0.86); 0.3 represents a trainee treating about one day per week.

Four results follow. First, the scenario ranking and the qualitative conclusions do not depend on the trainee capacity: Scenario C closes the median care gap in every variant (from 2030 at a factor of 0.3, from 2028 at 0.7 and above), whereas Scenario A closes it in none (minimum gap 262,000 persons/year even at 0.9). Second, the timing of the closure in Scenario B is sensitive to the assumption: at a fixed factor of 0.3 or 0.5, the median gap in Scenario B does not close at all (minimum 46,000 and 29,000 persons/year); at 0.7, it closes by 2033; at 0.9, by 2031; and in the funding-dependent main model, by 2032—the closure in the partial-funding scenario therefore hinges on the capacity per trainee rising above today’s part-time level once trainee services are funded, which is precisely the mechanism the survey data suggest (82% of trainees plan to expand their hours). Third, the effect on cumulative costs is moderate relative to the scenario differences (Table 8). By 2040, the fixed-factor variants hardly differ because the old-system trainees have since completed and only the phase-3 stock of the new system remains. Fourth, the counterfactual without any insurance funding of trainee services marks the lower bound of the lever: cumulative costs rise to €96/31/3.9 billion (A/B/C), the gap in Scenario B never closes, and Scenario C closes it only by 2032 instead of 2028 (Table 8). The window of need coverage in the partial-funding scenario therefore exists only because trainee services become insurance-funded.

#### 4.9.3. Additional Robustness and Decomposition Analyses

**Weekly-hours anchor.** Because potential capacity scales linearly with the weekly-hours anchor, the full simulation was re-run with the anchor set to 15.8 and 13.3 treatment hours/week (the activity-adjusted weighted means for both contracted and all listed practitioners from the authors’ weighted secondary analysis of the individual-level data of the ÖBVP member survey from December 2024 [9]; Section 5.10 and Supplementary Material S1.7), with the capacity triangle and the trainee module rescaled consistently. Under 15.8 h/week, the median care gap no longer closes in Scenario B (minimum ≈ 132,000 persons/year) and closes in Scenario C only from 2031 onward; under 13.3 h/week, it no longer closes, even in Scenario C (minimum ≈ 5500 persons/year in 2033; 66,458 in 2040), and cumulative follow-on costs rise to €103.7/39.5/7.5 billion and €113.5/53.2/15.7 billion (A/B/C) (Table S9). These runs hold the scenario activation shares constant across anchors (a one-factor sensitivity). Because the status-quo activation is calibrated on the observed reach, Scenario A falls below that reach at the lower anchors, and its cumulative costs there overstate the status-quo case; with the status-quo activation and full-funding shares rescaled by 21/h, so that Scenario A reproduces the accessible capacity of the 21-h main model, its cumulative costs are €86.5 billion (15.8 h/week) and €88.0 billion (13.3 h/week). The 21-h results should therefore be read as upper bounds on capacity (Section 5.10).

**Treatment intensity at dynamically projected capacity.** The static robustness matrix (Section 4.9.1) evaluates the 2023 capacity anchor. Applying the four treatment intensities to the dynamically projected accessible capacity (medians) of 2030 and 2040 yields, for a 9% need, coverage of 104%/113% in Scenario C at 13 units/patient/year but only 45%/49% at 30 units/patient/year (Table S10). The closure of the median care gap under Scenario C at 13 units/patient/year and the low coverage at higher intensities are thus the same model logic evaluated at different treatment intensities and thus not a contradiction.

**Separating the two trainee mechanisms.** Holding capacity per trainee fixed at the unfunded level f_0_ while making trainee services financially accessible isolates the pure accessibility effect: cumulative follow-on costs in Scenario B fall from €31.0 billion (no trainee funding; Table 8) to €15.7 billion compared to €13.1 billion in the main model, but the median gap then no longer closes completely (minimum ≈ 10,600 persons/year). The accessibility of existing trainee services thus carries most of the cost effect, whereas the complete closure of the Scenario B gap in 2032–2036 depends on the funding-induced rise in capacity per trainee from f_0_ toward f_1_ (Table S11).

**Scenario-independent chronification and waiting-time variants.** The three variants of Table 1 are implemented as separate runs in which identical chronification and waiting-time parameters apply to all three scenarios, while all policy parameters remain scenario-specific. The difference in cumulative follow-on costs between Scenarios A and C then ranges from €51.6 billion (conservative) over €62.2 billion (realistic) to €76.0 billion (pessimistic), as compared with €82.1 billion in the main model (Table S12). The scenario-specific coupling of the clinical–economic parameters thus accounts for roughly €20 billion of the A–C difference; the ranking of the scenarios is unaffected.

**Sequential decomposition of the policy levers.** Starting from Scenario A and cumulatively switching parameter groups to their Scenario C distributions (common random numbers) shows that activating the capacity of listed psychotherapists carries the largest single contribution: median cumulative follow-on costs fall from €83.4 to €3.5 billion, and the median gap closes by 2030. Insurance funding of trainee services advances closure to 2028 (−€1.4 billion); the training-capacity group raises the accessible capacity from ≈754,000 to ≈905,000 persons/year without further affecting the gap at 13 units/patient/year; and the clinical–economic parameters contribute the remaining −€0.8 billion (Table S13). Because the gap already closes at the first step under 13 units/patient/year, this ordering partly reflects the sequential design; at higher treatment intensities and after the 2038 transition, the capacity contribution of the training levers becomes decisive (Section 4.9.1 and Section 4.5).

#### 4.9.4. Key Conclusions of the Robustness Analysis

The robustness analysis sharpens the model’s main message in an important respect. At the current treatment intensity of 13 units per patient per year, activation is the dominant lever for closing the care gap. In the higher-intensity scenarios (30–40 units), the finding changes substantially: coverage of a 9% need in Scenario C falls from about 68% at 13 units/year to about 30% at 30 units/year at the 2023 capacity anchor (Figure 6) and remains below 50%, even with the projected 2040 capacity (Table S10), so that additional capacity expansion becomes indispensable. The trainee-capacity assumption, by contrast, changes the timing of the gap closure in Scenario B but not the scenario ranking (Section 4.9.2). Lower weekly-hours anchors shift the conclusion in the same direction, even at 13 units/patient/year (Section 4.9.3, Table S9).

The policy consequence: Activation and funding policy remains the most important short-term lever—particularly because, once the legal and contractual conditions are in place, it takes effect without training latency. In the medium to long term, however, the model suggests that it would need to be complemented by substantial capacity expansion (more master’s places, more phase-3 places, more outpatient clinics) as treatment intensity moves from 13 units towards higher treatment intensities of 30+ units/patient/year.

## 5. Discussion

### 5.1. The Central Conceptual Shift: Activation as a Necessary but Not Sufficient Lever

The most fundamental result of this study is conceptual in nature and is further differentiated by the robustness analysis: the FTE calibration, with the empirical anchors of Dinhof et al. [2] and Gruber et al. [3], suggests that Austrian psychotherapeutic care—at the current treatment intensity of 13 units per patient per year—suffers primarily from insufficient activation of existing capacity and less from a shortage of psychotherapists in the narrow sense. This diagnosis changes, however, in the higher-intensity scenarios: at 30–40 units/year, activation becomes necessary but not sufficient; additional capacity expansion is therefore structurally required.

Even allowing for part-time work, private practice, and self-pay priorities, the contrast between the computed capacity and the 199,250 persons reached with partial or full funding in 2022 (Section 2.2) suggests a substantial activation gap. The simulation indicates that, even with a stagnating profession, doubling the activation rate—from about 30% to about 60%—could markedly reduce the care gap, provided treatment intensity remains constant.

One policy implication of the model results—under the stated assumptions—is that a strategy relying exclusively on expanding training places, without addressing the funding and structural side in parallel, could end in an activation trap: growing therapist numbers without correspondingly growing socially accessible care. Both levers are complementary and necessary.

### 5.2. The Dual Role of Supervised Trainees (i.A.u.S./PiFALS)

Persons in training under supervision (2799 in 2023 [9]; 3238 in 2025 [14]) occupy a system-critical dual function: they are simultaneously part of the current care capacity and the future workforce. A lack of funding for their services therefore acts as a damage multiplier on three levels: it withholds current care (unfunded trainees cannot deliver insurance-funded services in outpatient clinics), slows training (through self-funding pressure), and erodes the institutional base (outpatient clinics without trainee funding are not economically viable).

The model quantifies this lever for the first time. At the survey-anchored treatment capacity of 60% of a fully active therapist in the current unfunded state (Table S7), the roughly 3400 trainees of 2026 represent about 147,000 persons/year of potential capacity, 18% of the national total, rising to about 270,000–350,000 persons/year (25–30%) by 2032 when the old-system stock peaks; the phase-3 stock of the new system has built up and the capacity per trainee has risen with the phased-in funding. Funding their services activates this capacity within one to four years without a single additional licensure (Section 4.1). The survey data add a second mechanism to this lever: trainees today work part-time in psychotherapy (Section 3.8.2), so insurance funding not only activates existing services but raises the services per trainee—in the model, from 0.60 to up to 0.90 of a fully active therapist—and the robustness analysis shows that the closure of the gap in Scenario B depends on this rise (Section 4.9.2). Without insurance funding of trainee services, the gap in Scenario B would not close in any year (Table 8). Insurance funding of trainee services is accordingly a lever that acts on both sides of the dual role at once and, in the model, within one to four years; however, under the current law, trainee services are not an insurance benefit (Section 2.3). Its effect is, however, time-limited by the transitional law: the old-system trainees complete by 2038, and the lever then depends entirely on the size of the new system’s phase-3 stock.

### 5.3. The Reform Dip and the Transition Cliff of 2038

Two transition effects need to be separated. The classical ‘reform dip’—a temporary shortfall of licensures between the end of the old pathway and the ramp-up of the new—is, in the model, short and shallow: master’s entrants are licensed from 2031, the old pathway continues to deliver about 714 completions per year until 2038, and the licensed stock grows in all scenarios until then. The consequential effect is the transition cliff of 2038 created by § 60 PThG 2024: within two years, the old pathway’s completions and its supervised trainees (about 4200 at the 2032 peak) disappear; from 2039, the licensed stock shrinks, the trainee capacity falls to about a fifth to a third of its peak; and, in Scenario B, the closed care gap reopens (Section 4.5). The FTE calibration nevertheless shows that the care effect of this cliff is far smaller than its workforce effect wherever activation is high: in Scenario C, the gap stays closed with a 99% probability in 2040; in Scenario A, it widens by about 34,000 persons/year—not primarily because of the cliff but because activation remains low.

This is an important finding for health-policy prioritization: the reform dip of the early 2030s has been overestimated in the debate so far. At the same time, two other quantities have been underestimated—the short-term activation and funding question and the throughput of the new system (master’s places, phase-3 places) needed after 2038 in the long term (Section 4.8).

### 5.4. Psychotherapeutic Outpatient Clinics as a Central Structural Component

In the FTE model, psychotherapeutic outpatient clinics emerge as a central structural component for the activation question. They combine phase-3 training capacity with care provisions in one integrated institution and enable trainee services to be publicly funded—thus, they become simultaneously care effective and training supportive. The model makes the phase-3 place a binding constraint in the status quo scenario: any expansion of the master’s intake without additional places lengthens the queue rather than the workforce (Section 4.8); in Scenarios B and C, the places suffice for the current master’s intake but not for an expanded one. Compared with physician-led psychosocial centers, which primarily offer psychiatric care with subordinate psychotherapy, psychotherapeutic outpatient clinics address the specific structural gap between the training and care systems.

### 5.5. The Sensitivity of the Retirement Rate: A Model-Based Clarification

Within the scenarios, the retirement rate correlates only moderately with cumulative follow-on costs but strongly with the licensed stock and accessible capacity in 2040 (Section 4.7). In the health-policy debate, the retirement wave is often portrayed as the central risk factor. The FTE-calibrated simulation shows a more differentiated picture, namely retirements act via the stock → potential capacity → accessible capacity → care gap → costs, and with each retired therapist removing more accessible capacity, the higher the activation rate becomes; in the underfunded Scenario A, most potential capacity is not activated in the first place, so retirements hardly show in the cost balance. Moreover, until 2038, completions exceed retirements in every scenario, so the stock grows despite the aging profession; the retirement rate becomes decisive only after the transition cliff, when the new system’s throughput has to cover it alone.

This does not mean retirements are unimportant. Their significance lies more in the capacity base, in the post-2038 dynamics, and in demographic time-criticality than in the absolute level of follow-on costs. Methodologically, this teaches us not to confuse sensitivity analyses with importance analyses: a parameter can be structurally time-critical without appearing dominant in the sensitivity correlation.

### 5.6. Long-Term Economic Effects and the Secondary-Cost Anchor

The modeled cumulative difference in follow-on costs of roughly €82 billion (Scenario A minus Scenario C, 2026–2040) corresponds to an average annual difference of about €5.5 billion—about 1.1% of Austrian GDP (€494 billion in 2024). These figures denote avoided downstream societal costs and not net savings: the incremental implementation costs of Scenarios B and C—additional insurance expenditure for psychotherapy, outpatient-clinic expansion, remuneration of supervised trainees, additional master’s and phase-3 training places, and supervision—are outside the model’s scope and would have to be netted against the avoided costs in a full economic evaluation. This order of magnitude is consistent with European estimates: the OECD and the European Commission put the total cost of mental ill-health at more than 4% of GDP across the 28 EU countries—over €600 billion in 2015—of which about 1.6% of GDP are indirect labor-market costs and 1.3% direct health expenditure [18].

The cross-check against Austrian secondary-cost data (Section 3.11, Table 4) indicates that the model’s per-person cost parameters are low relative to this clinically selected high-utilizer population: observed per-patient costs in the high-utilizer population studied by Seitz et al. [16] exceeded the model’s per-person cost assumptions (€5000–18,000 in total) by one to two orders of magnitude. Because this comparison population is a clinically selected high-utilizer group and not directly representative of the heterogeneous population constituting the care gap, these figures do not establish a strict lower bound; the finding nevertheless replicates, at the micro level, the macro-level thesis of this study: expenditure on psychotherapeutic treatment is small relative to the secondary costs it avoids.

### 5.7. International Contextualization of the Activation Gap

International benchmarking of the Austrian activation gap is methodologically difficult because few countries systematically publish comparable data on computed care capacity (FTE) and socially accessible care. Nevertheless, the literature offers structural indications. Germany: In the German statutory system, with needs-based planning performed by the regional associations of statutory health insurance physicians, care is steered via practice seats. The 2017 structural reform was intended to shorten waiting times. Still, a practice-based cohort study found the average wait for a first consultation unchanged at about three weeks, and the interval between first contact and the start of treatment increased from about 18 to 20 weeks [19]; this indicates a likewise relevant activation gap albeit differently structured: the bottleneck is less the activation of existing capacity than the cap on insurance-funded seats. Switzerland: With the change to the prescription model on 1 July 2022, psychological psychotherapy is publicly funded on medical prescription rather than under delegation. Official monitoring shows how quickly a structural reform propagates: expenditure on psychological psychotherapy rose from CHF 528 million (2021) to an estimated CHF 922 million (2024), i.e., by about 20% per year, while the supplied full-time equivalents grew by roughly 35% between mid-2022 and mid-2023 [20]. The Netherlands: The Dutch system combines mandatory health insurance with stepped care levels (basis-GGZ, specialist GGZ). It achieves comparatively broad access, albeit under strict indication rules and capped session numbers [21]. The United Kingdom (IAPT/NHS Talking Therapies): The Improving Access to Psychological Therapies program (since 2007; NHS Talking Therapies from 2023) set out to train more than 10,500 new therapists and, by 2018, was treating over 560,000 patients per year, with routine outcome data obtained for 98.5% of them [22]; what is methodologically interesting is its combination of workforce expansion (tiered therapist qualifications) with a high activation—and yet the typical treatment duration is short, which, in the terms of our model, corresponds to a low treatment intensity.

The international comparison suggests that Austria’s activation gap falls within a range typical of mixed systems but is structurally unfavorable. Structural reforms, such as the Swiss prescription model or the British IAPT, show that a substantial increase in the activation rate is possible within a few years, each with specific trade-offs: in Switzerland, there was a strong cost increase; in the UK, there was a reduction in treatment intensity. An Austrian reform would have to address these trade-offs explicitly.

### 5.8. On the Operationalization of Need

Using 9% of the population as the upper need estimate (mode 7.5% in the triangular distribution) follows the treatment-willingness operationalization by Dinhof et al. [2]. This quantity differs conceptually from epidemiological need (12-month prevalence of mental disorders requiring treatment, typically estimated at 15–20% in Austria) and from actual care demand (clinical indication × treatment motivation × availability).

The robustness analysis across 5%, 7.5%, 9%, and 12% need (Section 4.9) shows that the qualitative scenario ranking is stable across all need levels. The absolute care gap and coverage, however, are highly sensitive: at 5% need, Scenario C could computationally exceed 100% coverage at current intensity, whereas at 12%, this was only about 51%. The choice of need operationalization is therefore a politically and scientifically important prior decision that must be made transparent.

### 5.9. Policy Implications

The simulation results suggest the following conditional policy implications; their estimated effects depend on the assumed parameterization and have not been prospectively validated. They are model-based hypotheses that require empirical validation. **(1) Activation as the time-critical lever:** At the current treatment intensity, activating existing capacity has the strongest short-term effect; in the higher-intensity scenarios, a parallel capacity expansion would be required. Within the model’s assumptions, this points to fair insurance tariffs (raised to professionally justified fee levels), statutory-insurance contracting with outpatient clinics (including trainee services once § 135 ASVG has been amended), regional care planning, and reduced bureaucratic access barriers. **(2) Insurance funding of supervised trainees’ services:** The model results suggest recognizing and funding trainee services as care services from the moment the register listing takes effect (1 October 2026), most directly by amending § 135(1)(3) ASVG and the corresponding provisions for other insured groups (Section 2.3); in the model, this funding is the lever that closes the care gap in the partial-funding scenario by 2032. **(3) Expansion of psychotherapeutic outpatient clinics with phase-3 capacity:** Outpatient clinics—as hybrid structures of care and training—are, within the model’s parameterization, a central structural component; the results suggest expanding these within spatial–regional care planning, endowed with publicly funded quotas, and certified as core institutions of phase-3 training. **(4) A monitoring system for FTE availability:** Austria so far lacks systematic reporting of treatment hours, age structure, and activation rates in psychotherapeutic care; we therefore recommend annual monitoring of psychotherapeutic care capacity by BMSGPK and/or GÖG that records not only the number of registered psychotherapists but also full-time equivalents, age structure, retirements, training capacity, the number of trainees in specialist training under teaching supervision, care sector, and funding status as preconditions for evidence-based workforce planning. **(5) Master’s and phase-3 capacity of the new system:** At the current master’s intake of about 1000 per year (about 500 publicly funded places at public universities and an estimated 500 at private universities), the new system’s licensures (about 580–840 per year) fall short of retirements after 2038 in every scenario, such that the licensed stock shrinks; about 1500 master’s places per year with proportionally growing phase-3 places at universities, outpatient clinics, and teaching practices are, in the model, the intake at which the workforce keeps growing after the transition cliff (Section 4.8)—phase-3 places without master’s places are as ineffective as master’s places without phase-3 places, whose absence lengthens training through waiting times and reduces long-term output. **(6) Periodic workforce and care projections:** The present model could serve as the basis for periodic (e.g., biennial) updated projections as further primary data become available. **(7) Insurance expansion as a demand-side measure:** A substantial increase in publicly funded psychotherapy quotas would be the most direct form of activation increase and could simultaneously reduce social inequality in access to care.

### 5.10. Limitations

The model has several substantial limitations that must be discussed explicitly. **The FTE anchor is an empirical median:** The 21 treatment hours/week [2] are an empirical median, not a legally defined full-time equivalent; actual variability in the profession is considerable (part-time work, multiple employment, private practice); the model maps the median assumption, not the distribution. A weighted secondary analysis of the individual-level data of the ÖBVP member survey from December 2024 [9] (listed practitioners only, post-stratified to federal-register margins; Supplementary Material S1.7) yields, for the same profession, an activity-adjusted weighted mean of 13.3 h/week across all listed practitioners and 15.8 h/week among contracted practitioners. The two figures do not measure the same construct—a median against a weighted mean and elicited treatment hours against treatment time isolated from a reported activity mix—and neither is a correction of the other. Because potential capacity scales linearly with this parameter, the capacity levels reported here should be read as upper bounds until the two instruments are reconciled: under the lower anchors, the median care gap no longer closes in Scenario B (15.8 h/week), nor at 13.3 h/week, even in Scenario C, and the cumulative follow-on costs rise accordingly (Table S9). **Treatment intensity of 13 units/patient:** This figure reflects the current value [3] and presumably underestimates the treatment intensity required for many clinical pictures; assuming 30 units/patient, theoretical capacity falls to about 290,000 persons/year—a different and more realistic magnitude of the activation gap. **Chronification modeling:** Despite the three-variant approach, quantitative chronification dynamics by waiting duration remain a central uncertainty; empirically validated risk curves are urgently needed. **No regional differentiation:** The model operates at national level; considerable regional differences (Vienna versus rural federal states; care density, transport links, outpatient-clinic density) are not represented. **Quality dimensions:** the model maps capacities quantitatively, not qualitatively; it does not consider modality-specific fit, treatment quality, differential effectiveness, or indication decisions. **No system-adaptation dynamics:** The model assumes that actors (patients, therapists, institutions) do not react to the evolving care gap; in reality, price, substitution, and political adjustment effects would occur. **Sensitivity analysis as a correlation measure:** The within-scenario rank correlations in Figure 5 capture monotonic relationships but no interaction effects, and they describe parameter uncertainty within a policy package, not the effect of choosing between packages; extended variance-based sensitivity analyses (Sobol indices, EFAST) would be useful in follow-up work. **Trainee capacity:** The treatment capacity of supervised trainees in the current unfunded state (60%, range 50–70% of a fully active therapist) is anchored on a stratified re-analysis of a single unweighted member survey, with a 43.5% response rate, categorical answers converted to category midpoints, and respondents who are younger and more active than the profession as a whole [9] (Table S7); its rise to 90% (range 80–100%) under full insurance funding, the one-year lag, and the equal treatment of old-system and phase-3 trainees are expert assumptions of the authors. Because the trainee line contributes 18% of potential capacity in 2026 and up to 30% in 2032, this assumption sets the trainee effect level and the timing of gap closure in Scenario B (Section 4.9.2). A longitudinal measurement of trainees’ treatment hours before and after the register listing of 1 October 2026 is the single most important calibration task (Section 5.11). **Training pipeline:** The master’s intake (500 public places as a ministerial planning figure, 500 private places as an estimate), the direct entrants under § 10, the phase-3 duration and the lead time to full trainee activity are planning assumptions rather than observed values; the accelerated completion of all old-system candidates by 2038 follows the law and not observed behavior; the queue model assumes no drop-outs while waiting; and the propaedeutic candidates who switch to the master’s program are assumed to compete for the existing places. **Need operationalization:** The 9% treatment-willingness value is based on [4] and is a model assumption; actual clinical need, epidemiological need, and treatment willingness can diverge considerably. **Secondary-cost anchoring:** The cross-check against Austrian secondary-cost data (Table 4) relies on a clinically selected high-utilizer population [16] and on aggregate statistics with reference years 2006–2016; it indicates that the cost parameters are low relative to this specific population but is not a formal cost-of-illness validation for the average person in the care gap.

### 5.11. Future Research

The present simulation identifies several priority research needs: an empirical FTE survey with full coverage and distribution analysis (not only the median) of weekly treatment hours in the Austrian profession and of persons in training under supervision, extending the cross-sectional survey re-analysis of Table S7 into a panel that observes trainees before and after the register listing of 1 October 2026; differentiated measurement of activation rates by sector (insurance quota, self-payment, outpatient clinic, teaching practice); validation of the chronification–waiting time relationship in Austrian data (longitudinal study); measurement of actual waiting times by care sector and region; a cost analysis of specialist training under teaching supervision (cost per training place, funding requirements, economic viability of outpatient-clinic structures); an evaluation study of existing psychotherapeutic outpatient clinics in Austria (care quality, cost efficiency, training integration); system-dynamics modeling with explicit feedback loops (price–quantity effects, substitution dynamics); extended variance-based sensitivity analyses (Sobol indices); a comparative study of activation rates and funding models in Germany, Switzerland, and the Netherlands; and an analysis of regional care density and its FTE activation rates.

## 6. Conclusions

The present FTE-calibrated simulation model suggests a differentiated finding: at the current treatment intensity of 13 units per patient per year, psychotherapeutic care in Austria is limited primarily by the insufficient activation of existing capacity into socially accessible, publicly funded care and less by a shortage of psychotherapists in the narrow sense. The computed personnel capacity of about 669,000 persons/year contrasts with fully funded care of only about 96,500 persons (2022). The robustness analysis sharpens this finding for higher treatment intensities: in the higher-intensity scenarios of 30–40 units per patient per year (adequate intensity varies with diagnosis, severity and treatment modality), activation becomes necessary but is not sufficient; additional capacity expansion is therefore structurally required. Fewer weekly treatment hours have the same effect: because capacity scales linearly with the hours each therapist treats per week, the survey-based anchors of 15.8 and 13.3 treatment hours/week instead of 21 act like about 17 and 21 units/patient/year at the 21-h anchor, and the median care gap then no longer closes in Scenario B, nor at 13.3 h/week, even in Scenario C (Section 4.9.3, Table S9); the reported capacity levels are therefore upper bounds.

The central health-policy thesis of the model can be stated as follows: investments in public funding, in psychotherapeutic outpatient clinics, in the insurance funding of supervised trainees’ services, and in master’s and phase-3 capacities are, in the scenario comparison, structural measures with high leverage on access and avoided downstream societal costs, whose implementation costs lie outside the model’s scope (Section 5.6)—with the activation dimension dominant in the short term and parallel capacity expansion becoming necessary in the medium to long term as treatment intensity rises.

In the most favorable scenario (expansion/full funding) and under the 21-h weekly-hours anchor, the care gap can be closed by 2028, with avoided follow-on costs of roughly €82 billion versus the status quo by 2040. Even in the moderate scenario (partial funding), the gap closes from 2032 to 2036 and is reduced by about 88% by 2040, with avoided follow-on costs of about €70 billion—in both cases largely by funding the services of trainees who already treat under supervision who today mostly work part-time and have capacity to spare. This trainee-capacity line rests on a single unweighted member survey and on expert assumptions about capacity under full funding; it is the first calibration priority once post-reform data become available. The reform dip of the early 2030s is shallow in the FTE model; the decisive transition effect is the cliff of 2038, when the old pathway’s completions and trainees disappear and the new system’s throughput—about 1000 master’s places per year today (about 500 publicly funded places at public universities and an estimated 500 at private universities), and about 1500 needed with matching phase-3 places—decides whether the workforce keeps growing. The methodological lesson of this study is that workforce models without FTE calibration and without three-level capacity differentiation miss the actual health-policy lever.

Beyond care provision, these findings also inform national research and training policy. The Austrian University Development Plans (GUEP 2025–2030 and GUEP 2028–2033) [23,24] do not address psychotherapy specifically, but they set the framework objectives—interdisciplinary competence, lifelong learning and international collaboration—within which the new university-based psychotherapy training will have to be situated; this perspective should also inform the reading of OECD indicators that portray Austrian outpatient care as capable of improvement.

Methodologically, the reported figures are scenario simulations under explicit assumptions and not empirical forecasts. Their main function is to structure the problem, identify critical decision points, and estimate orders of magnitude. The most urgent next research step is to deepen the calibration—particularly regarding activation dynamics, the chronification–waiting time relationship, and regional care. Austrian psychotherapeutic care stands at a reform-driven crossroads; decisions in the coming years—especially the funding structure—will determine the care situation up to 2040 and beyond.

## Figures and Tables

**Table 1:** Three-variant modeling of the waiting time–chronification relationship.

Variant	Assumption on the Waiting Time–Chronification Link	Model Parametrization
Conservative	Small risk increase; linear rise after a threshold	Waiting-time factor: 1.0–1.3; base risk: 10%
Realistic	Moderate risk increase; own scenario assumption, direction motivated by [5]	Waiting-time factor: 1.0–1.8; base risk: 18%
Pessimistic	Strong risk increase; own scenario assumption, direction motivated by [6]	Waiting-time factor: 1.0–2.5; base risk: 28%

Note: The main model draws the chronification and waiting-time parameters from scenario-specific distributions (Table A2); the three variants of this table are implemented as scenario-independent robustness runs in Section 4.9.3 (Table S12).

**Table 2:** Empirical model anchors for the FTE calibration.

Parameter	Value	Source
Psychotherapists in the professional register (2023)	11,676	[2,9]
Of whom are under 65 years	8523 (73%)	[2]
Of whom are over 65 years	3159 (27%)	[2]
Median treatment activity	21 h/week	[2]
Working weeks per year	45	Model assumption
Annual treatment hours per therapist (FTE)	945 h	21 × 45
Current units per patient per year	13	[2]
Theoretical capacity per therapist aged under 65	72.7 persons/year	945/13
Theoretical total capacity, Austria	≈669,121	[2]
Persons reached with partial/full funding (2022)	≈199,250	[3]
Persons reached with full funding (2022)	≈96,500	[3]
Need/treatment willingness	≈9% of the population	[4]
Psychotherapy education completions (Fachspezifikum; 2024/25)	624	[14]
Persons in psychotherapy education (Fachspezifikum; 1 June 2025)	5601	[14]
Persons in training under supervision (i.A.u.S.; 1 June 2025)	3238 (57.8%)	[14]
Master’s places per year, new system (from 2026/27)	≈1000 (500 public + ≈500 private)	Planning figure (BMBWF)/authors’ estimate
Capacity per supervised trainee (share of a fully active therapist; both systems)	0.60 unfunded (today) → 0.90 fully insurance-funded	Survey re-analysis (Table S7) [9]/expert assumption (authors’ own)
Monte Carlo iterations	30,000	Model parameter

Note: FTE = full-time equivalent. i.A.u.S. = in training under supervision (old system); PiFALS = psychotherapists in specialist training under teaching supervision (Psychotherapeut:innen in Fachausbildung unter Lehrsupervision; new system); these are combined together as ‘supervised trainees’. Need is operationalized as treatment willingness × population.

**Table 3:** Comparison of the reform cohorts by key characteristics.

Characteristic	Old System (PThG 1990)	Master’s-Entry Cohorts (PThG 2024, from 2026/27)	Bachelor’s–Master’s Route (PThG 2024)
Age at entry	Mean 32–38 years	Variable (prior bachelor’s-equivalent studies)	Mean 22–26 years
Training duration	Turnover of the pool ≈ 9 years; completion by 30 Sept 2038	2 years for master’s + 2–5 years for phase 3 (+ waiting time for a place)	5 years BSc/MSc + 2–5 years phase 3
Training path	Propaedeutic + Fachspezifikum	Credit transfer → MSc + specialist training under teaching supervision	BSc + MSc + specialist training under teaching supervision
Age at professional entry	Mean 40–45 years	Variable	Mean 29–33 years
Expected professional years	15–25 years	Variable	30–40 years
Output by 2040	≈714/year until 2038 → 0 from 2039	576 (A)–838 (C) per year from 2033	Bachelor’s graduates fill the same master’s places (no separate output)

Note: Output figures are Monte Carlo medians; the range for the master’s-entry cohorts spans Scenarios A and C. In Scenario A, the output is limited by phase-3 training places (about 1050 candidates waiting in 2040).

**Table 4:** Cross-check of the model’s cost parameters against Austrian secondary-cost data (compilation by Seitz et al. [16] for a high-utilizer population with somatic symptom disorder; reference years 2006–2016).

Cost Component	Model Assumption (Mode)	Austrian Empirical Anchor	Source
Base cost per untreated person/year (excess GP, emergency, medication use)	€5000	€1093/patient/year for examinations, consultations, GP changes and medications alone (t1: €412 + €274 + €29 + €378)	[16]
Chronification excess cost/year	€10,000	Hospitalization costs €115,252/patient/year (t1) at €3168/hospital day; six-month excess costs of €6123–€31,883 by severity level (2019 euros)	[8,16]
Productivity loss per person/year	€3000	Sick-leave costs €31,094/patient/year (t1) at €186/sick-leave day (≈35 million workdays lost/year, ≈€6.5 billion in total)	[7,16,17]
Sick-leave days (model: 24 days/year)	24 days/year	64.5 sick-leave days/patient/year avoided after integrated treatment	[16]
Total secondary costs, high-utilizer population	—	€147,689 → €67,943/patient/year; reduction €79,746, i.e., to 46% of baseline, one year after integrated care	[16]

Note: t1 = year before the intervention; t2 = year after the intervention. For the hospitalization and sick-leave components, the model’s per-person cost parameters (€10,000/€3000; modes) are one to two orders of magnitude below the observed per-patient secondary costs in high-utilizer populations [16]. For the base-cost component, the direction is reversed: the empirical anchor of €1093 covers examinations, consultations, and medication only and is therefore narrower than the ambulatory excess utilization represented by the model’s €5000. The empirical anchors refer to a clinically selected population and are not directly transferable to the average untreated person in the care gap; they serve as an upper-bound plausibility check.

**Table 6:** Sensitivity analysis: Numerical correlation values and interpretation.

Parameter	ρ with Cumulative Costs 2040 (A/B/C)	ρ with Accessible Capacity 2040 (A/B/C)	Interpretation
Need (% of the population)	+0.70/+0.74/+0.79	0.00/0.00/0.00	Dominant within-scenario cost uncertainty; weight increases with the activation level; no effect on capacity
Access/funding activation (therapists)	−0.26/−0.47/−0.40	+0.66/+0.66/+0.48	Strongest steerable parameter for both endpoints
Capacity per therapist (FTE anchor: treatment hours/week)	−0.16/−0.28/−0.37	+0.39/+0.39/+0.46	Gains weight as activation rises: every FTE hour counts more once it is funded
Waiting-time factor	+0.21/+0.04/+0.01	0.00/−0.01/0.00	Cost driver only in the underfunded scenario
Retirement rate	+0.11/+0.15/+0.06	−0.47/−0.45/−0.52	Moderate for costs, strong for capacity and dominant for the licensed stock (ρ ≈ −0.8)
Insurance funding of trainee services	−0.11/−0.14/−0.01	+0.14/+0.09/+0.04	Immediate lever; its level effect (gap closure in B and C) exceeds its dispersion effect
Chronification rate	+0.12/+0.05/+0.02	−0.01/−0.01/0.00	Cost multiplier while a gap persists
Full-funding share	+0.01/−0.01/−0.10	0.00/0.00/+0.14	No direct effect on costs, gap or accessible capacity by construction (equity indicator, level 3); the correlations in Scenario C arise because the consistency constraint (full-funding share ≤ activation rate, Section 3.3) couples it to the activation rate
Lead time to full trainee activity	+0.03/+0.06/+0.01	−0.06/−0.11/−0.15	Delays the phase-3 contribution
Master’s places, private universities	−0.04/−0.05/0.00	+0.18/+0.23/+0.31	Pipeline parameter; visible in capacity and stock, not in costs
Completion rate, new system	−0.03/−0.05/0.00	+0.17/+0.19/+0.20	Pipeline parameter; acts with a lag
Admissions, old system (until 2030)	−0.03/−0.03/−0.02	+0.06/+0.05/+0.06	Small; all admitted candidates complete by 2038
Capacity per trainee, unfunded (f_0_)	−0.02/−0.02/−0.02	+0.02/+0.01/+0.01	Small dispersion effect; sets the level of trainee capacity before funding takes effect (survey anchor, Table S7)
Capacity per trainee, fully insurance-funded (f_1_)	0.00/−0.02/0.00	+0.01/+0.02/+0.02	Small dispersion effect; acts only with the phased-in funding (expert assumption)
Direct entry to phase 3 (§ 10)	−0.01/−0.02/0.00	+0.06/+0.07/+0.07	Small numbers (20–100 per year)
Phase-3 duration	+0.01/−0.02/+0.01	+0.04/+0.09/+0.13	Longer phase 3 = larger treating trainee stock, later licensure
Phase-3 training places	−0.01/+0.01/0.00	+0.07/−0.01/0.00	Binding in Scenario A only (stock 2040: ρ = +0.33 in A)

Note: Spearman rank correlations between parameter values and two endpoints—cumulative follow-on costs by 2040 and accessible capacity in 2040—computed within each scenario (*n* = 30,000 per scenario); the largest absolute cost correlation across scenarios orders the rows. Table S5 reports correlations with licensed stock in 2040. f_0_/f_1_ = capacity per supervised trainee (both systems) in the unfunded/fully insurance-funded state (Section 3.8.2). Source: Authors’ simulation.

**Table 7:** Care gap, licensed stock, and waiting queue in 2040 as a function of the annual master’s intake (phase-3 training places scaled in proportion to the intake).

Master’s Places per Year (Medians, 2040)	Scenario A (Status quo/Underfunding)	Scenario B (Transition/Partial Funding)	Scenario C (Expansion/Full Funding)
1000 (current intake): care gap (persons/year)	320,254	37,043	0
1000: licensed psychotherapists	15,369	16,759	17,456
1000: waiting for a phase-3 place (persons)	1060	0	0
1500: care gap (persons/year)	269,460	0	0
1500: licensed psychotherapists	17,431	19,356	20,405
1500: waiting for a phase-3 place (persons)	1250	0	0
2000: care gap (persons/year)	218,436	0	0
2000: licensed psychotherapists	19,480	21,954	23,356
2000: waiting for a phase-3 place (persons)	1446	0	0
2500: care gap (persons/year)	167,664	0	0
2500: licensed psychotherapists	21,525	24,553	26,307
2500: waiting for a phase-3 place (persons)	1647	0	0

Note: Master’s intake fixed at the stated value in all runs (the main run draws 500 + 250–750); all other draws were identical to the main run. With the phase-3 places of Table A2 held constant instead of scaled, the results at 2500 master’s places are: Scenario A—care gap 231,073, licensed stock 15,776, and waiting queue 12,249 (mean waiting time 3.5 years); Scenario B—stock 20,525 and queue 6947; and Scenario C—stock 24,927 and queue 1818. Data source: MC_FTE_Kalibriert_Masterplaetze (*n* = 30,000 per cell).

**Table 8:** Robustness of the main results to the capacity per supervised trainee: no insurance funding of trainee services, fixed funding-independent factors, and the funding-dependent main model (medians; A/B/C).

Capacity per Trainee (Share of a Fully Active Therapist) and Funding Variant	Cumulative Follow-on Costs 2040 (€ Billion)	Care Gap 2040 (Persons/Year)	Gap Closure: A Minimum Median Gap (Year); B and C First Year with a Closed Median Gap
No insurance funding of trainee services (factor 0.60, never activated)	95.8/31.0/3.9	349,176/82,490/0	332,766 (2038); not closed; 2032
Fixed 0.3	90.5/23.3/1.9	336,826/65,117/0	321,747 (2038); not closed; 2030
Fixed 0.5	86.9/18.1/1.5	328,875/53,727/0	310,265 (2035); not closed; 2029
Fixed 0.7	83.3/13.8/1.3	320,699/42,231/0	286,845 (2033); 2033; 2028
Fixed 0.9	79.6/11.4/1.1	312,868/30,896/0	262,028 (2033); 2031; 2028
Funding-dependent 0.60 → 0.90 (main model)	83.4/13.1/1.3	320,736/36,951/0	286,736 (2033); 2032; 2028

Note: ‘Never activated’ = the insurance-funding share of trainee services is set to zero in all three scenarios (trainees continue to treat privately at the unfunded capacity f_0_ and count as potential, but not as accessible, capacity). Fixed factors apply to trainees in both training systems for each year and do not respond to insurance funding of trainee services; the main model uses f_0_ ~ Δ(0.50; 0.60; 0.70) rising to f_1_ ~ Δ(0.80; 0.90; 1.00) with phased-in funding (Section 3.8.2). All other draws are identical to the main run. ‘Not closed’ = the median care gap remains above zero in every year during 2026–2040. Peak potential trainee capacity (persons/year, A/B/C): No insurance funding of trainee services (factor 0.60, never activated)—231,239/235,782/242,380; fixed 0.3—115,955/118,031/121,412; fixed 0.5—193,259/196,719/202,354; fixed 0.7—270,562/275,407/283,296; fixed 0.9—347,866/354,094/364,237; and funding-dependent 0.60 → 0.90 (main model)—268,415/310,708/349,358. Data source: MC_FTE_Kalibriert_Traineekapazitaet (*n* = 30,000 per cell).

## Data Availability

The original data presented in this study are openly available via the Open Science Framework (OSF) at https://doi.org/10.17605/OSF.IO/FZXRT. The deposit contains the annotated simulation source code (Codes S1 and S2), the results files (Tables S1–S6 and S8), and the aggregate survey re-analysis (Table S7); all random draws are reproducible via the fixed seed 20260622. We also include key results files within the Supplementary Materials, and Supplementary Material S3 documents the model architecture, empirical data anchors, verification and calibration checks, and annotated code excerpts. All empirical model inputs are published aggregate statistics cited in the manuscript except for the unfunded trainee-capacity anchor, which is derived from a stratified re-analysis of the authors’ own anonymous member survey (partly reported in [9]); the aggregate results of that re-analysis are provided in Table S7, while the individual-level responses are not deposited. No third-party raw data were used.

## Supplementary Materials

The following supporting information can be downloaded at: https://www.mdpi.com/article/10.3390/healthcare14213610/s1, Supplementary Material S1: Robustness Re-Calculation: Need and Treatment Intensity; Table S1: Robustheitsmatrix_Bedarf_Einheiten_Szenarien.csv (deterministic robustness matrix, 48 constellations); Table S2: MC_Robustheit_Bedarf_Einheiten.csv (Monte Carlo robustness summary with 80%/95% prediction intervals); Table S3: MC_FTE_Kalibriert_AllYears_ANNOTATED.csv (annual medians and prediction intervals of the main simulation, 2026–2040); Table S4: MC_FTE_Kalibriert_Sensitivitaet_within_ANNOTATED.csv (within-scenario Spearman correlations with cumulative costs); Table S5: MC_FTE_Kalibriert_Sensitivitaet_Zielgroessen_ANNOTATED.csv (within-scenario correlations with costs, accessible capacity and licensed stock—Table 6); Table S6: MC_FTE_Kalibriert_Masterplaetze_ANNOTATED.csv (master’s-intake analysis—Table 7); Supplementary Material S2: Stratified Re-Analysis of the ÖBVP Member Survey (December 2024): Treatment Activity of Trainees under Supervision versus Listed Psychotherapists (Table S7) [9]; Table S7: Table_S7_survey_reanalysis.csv (treatment hours, patients, occupation, expansion plans, and fees); Table S8: MC_FTE_Kalibriert_Traineekapazitaet_ANNOTATED.csv (robustness to the trainee-capacity assumption, Table 8); Code S1: montecarlo_psychotherapie_fte_annotiert_mit_quellen.py (annotated source code of the main Monte Carlo model; key excerpts are in Supplementary Material S3); Code S2: Robustness re-calculation script (need × treatment intensity). Table S9: Table_S9_Stundenanker_Sensitivitaet.csv (weekly-hours-anchor sensitivity, 21/15.8/13.3 treatment hours/week); Table S10: Table_S10_Intensitaet_dynamische_Kapazitaet.csv (treatment intensities applied to the dynamically projected accessible capacity, 2030 and 2040); Table S11: Table_S11_Trainee_Mechanismen_Trennung.csv (trainee accessibility vs. capacity-rise mechanism); Table S12: Table_S12_ChronWartezeit_Varianten.csv (scenario-independent chronification and waiting-time variants of Table 1); Table S13: Table_S13_Hebelzerlegung.csv (sequential policy-lever decomposition A→C); Figures S1–S4: Per-scenario coverage heatmaps and the absolute care gap by treatment intensity (static 2023 capacity anchor); Supplementary Material S3: Technical Documentation of the Monte Carlo Model (model architecture, empirical data anchors with source attribution, reproducibility and implementation, detailed methodological limits, verification and calibration checks, operationalization of the activation rate, numerical summary of the robustness matrix and annotated code excerpts).

## Abbreviations

The following abbreviations are used in this manuscript:

**Abbreviation**	**Meaning**
FTE	Full-time equivalent
PiFALS	Psychotherapists in specialist training under teaching supervision (Psychotherapeut:innen in Fachausbildung unter Lehrsupervision); phase-3 trainees of the new system (PThG 2024); combined with i.A.u.S. as ‘supervised trainees’
i.A.u.S.	In training under supervision (in Ausbildung unter Supervision); trainees of the old system (PThG 1990) entitled to treat under supervision
PThG 2024	Austrian Psychotherapy Act 2024 (Psychotherapiegesetz 2024)
PT	Psychotherapist
ÖBVP	Austrian Federal Association for Psychotherapy
GÖG	Gesundheit Österreich GmbH (Austrian National Public Health Institute)
BMSGPK	Federal Ministry of Social Affairs, Health, Care and Consumer Protection
ÖGK	Austrian Health Insurance Fund
PI	Prediction interval
MC	Monte Carlo
IAPT	Improving Access to Psychological Therapies (UK)
BMBWF/BMFWF	Federal Ministry of Education, Science and Research/Federal Ministry of Women, Science and Research (Austria)
GGZ	Geestelijke gezondheidszorg (Dutch mental health care)
NHS	National Health Service (UK)
OECD	Organisation for Economic Co-operation and Development

## Appendix A. Model Specification: Core Formulas and Scenario Parametrization

This appendix specifies the core model formulas (Appendix A.1) and the scenario-specific parameter distributions with their evidential status (Appendix A.2), i.e., the information required to follow and critically assess the model. The full technical documentation—including model architectures, empirical data anchors with source attributions, reproducibility and implementation, detailed methodological limits, verification and calibration checks, operationalization of the activation rate, and a numerical summary of the robustness matrix and annotated code excerpts—is provided in Supplementary Material S3.

### Appendix A.1. Core Formulas with Calculation Examples

**Table A1:** Model formulas with calculation examples.

Quantity	Formula	Example Value
Capacity per fully active therapist	h/week × weeks/year ÷ units per patient/year	(21 × 45)/13 = 72.69
Capacity per registered therapist (age-corrected)	theoretical total capacity ÷ registered therapists	669,121/11,676 = 57.31
Funding activation (actual, 2022)	partially/fully funded reached ÷ theoretical capacity	199,250/669,121 = 0.298
Full-funding share (actual, 2022)	fully funded reached ÷ theoretical capacity	96,500/669,121 = 0.144
Need modeling	population × Δ(0.05; 0.075; 0.09)	median ≈ 647,000
Workforce update	PT(t+1) = PT(t) + completions old + completions new − retirements − exits	see Figure 3
Potential capacity	PT(t) × K_reg + [i.A.u.S.(t) + PiFALS(t)] × K_trainee(t)	scenario-dependent; trainee share ≈ 18% (2026), ≈ 25–30% (2032)
Trainee capacity per person	K_trainee(t) = 72.69 × [f_0_ + (f_1_ − f_0_) × f_fund × ramp(t − 1)]	unfunded: 0.60 × 72.69 = 43.6; fully funded: 0.90 × 72.69 = 65.4 (modes); 2040: 0.70 (A) · 0.79 (B) · 0.86 (C) × 72.69
Simultaneous phase-3 places	places per year × phase-3 duration	A: 550 × 3 = 1650 (modes)
Accessible capacity	PT(t) × K_reg × activation rate + trainee capacity × f_fund × ramp(t)	activation A: 33% · B: 58% · C: 82%; f_fund A: 30% · B: 65% · C: 90%
Care gap	max(need − accessible capacity, 0)	see Figure 4
Follow-on costs (annual)	gap × (base cost × waiting-time factor + chronification rate × excess cost + productivity cost)	Σ → see Table 5

Note: K_reg = age-corrected capacity per registered therapist; K_trainee(t) = funding-dependent capacity per supervised trainee (identical for both systems); f_0_/f_1_ = capacity factor in the unfunded/fully insurance-funded state; f_fund = insurance-funded share of trainee services; ramp(t) = 0 (2026), 0.25 (2027), 0.5 (2028), 0.75 (2029), and 1 (from 2030); and Δ(a, m, b) = triangular distribution with minimum a, mode m, and maximum b.

### Appendix A.2. Scenario Parametrization (Triangular Distributions)

Table A2 documents the triangular distributions used per scenario in the form (minimum; mode; maximum). These values sit at the interface between model assumptions and simulation results and should be the focus of any critical review.

**Table A2:** Scenario-specific triangular distributions of the model parameters.

Parameter	A: Status quo	B: Transition	C: Expansion
Funding activation	(0.25; 0.32; 0.42)	(0.42; 0.58; 0.72)	(0.68; 0.82; 0.95)
Full-funding share	(0.10; 0.15; 0.22)	(0.25; 0.38; 0.55)	(0.60; 0.78; 0.90)
Completion rate, new system	(0.45; 0.60; 0.75)	(0.55; 0.70; 0.82)	(0.65; 0.80; 0.90)
Phase-3 training places (per year)	(350; 550; 800)	(800; 1200; 1600)	(1300; 1800; 2500)
Insurance-funded share of trainee services (target; phased in by 2027–2030)	(0.15; 0.30; 0.50)	(0.40; 0.65; 0.85)	(0.75; 0.90; 1.00)
Chronification rate	(0.22; 0.35; 0.50)	(0.13; 0.23; 0.34)	(0.06; 0.13; 0.22)
Waiting-time cost factor	(1.25; 1.70; 2.40)	(1.05; 1.25; 1.60)	(0.95; 1.05; 1.20)

Note: Values are (minimum; mode; maximum) of the corresponding triangular distribution. The following common distributions and constants apply across scenarios (model version 2.1): the need rate (0.05; 0.075; 0.09); capacity per registered therapist (50; 57.31; 68); capacity per supervised trainee relative to a fully active therapist (72.69 persons/year), identical for both training systems—unfunded state f_0_ (0.50; 0.60; 0.70), anchored on the survey re-analysis in Table S7, and fully insurance-funded state f_1_ (0.80; 0.90; 1.00), based on the expert assumption of the authors, with the capacity per trainee following the phased-in funding of trainee services with a one-year lag; admissions to the old pathway until 2030 (850; 950; 1100 per year) and turnover time of the old pool (8; 9; 10 years); drop-out share of throughput 12.7%; master’s places per year—500 (public universities, planning figure) + (250; 500; 750) (private universities); direct entrants to phase 3 (20; 50; 100 per year); phase-3 duration (2; 3; 5 years); lead time to full trainee activity (0.5; 1; 2 years); waiting candidates treat at 50%; base retirement rate (0.020; 0.035; 0.055); retirement growth (0; 0.015; 0.035); other exits (0.003; 0.007; 0.014); base cost per untreated person (€2500; €5000; €9000); chronification excess costs (€4000; €10,000; €20,000); and productivity costs (€1000; €3000; €7000). The parameters ‘new entries/training duration/phase-out of the old system’, ‘first-year students’, and ‘delay of the new system’ of model version 1 are replaced by these quantities. Evidential status of the distributions: empirically anchored (mode taken from published data)—need rate [2,4], capacity per registered therapist [2], f_0_ (Table S7), and admissions to and turnover of the old pathway [13,14]; official planning value—master’s places at public universities; expert assumptions of the authors—f_1_, master’s places at private universities, direct entrants, phase-3 duration and lead time, the 50% waiting-capacity share, completion rates, and the cost parameters (Section 3.10); empirically informed assumption—the retirement parameters (the age structure is known, individual exit behavior is not); and scenario-defining—the seven scenario-specific parameters of this table, with Scenario A anchored at the observed 2022 activation and full-funding values.
