# Supplementary material for: Psychotherapeutic Care in Austria 2026–2040: An FTE-Calibrated Simulation of Workforce, Funding and Care Capacity
Source: Healthcare (Basel). 2026 Oct 25;14(21):3610. doi: 10.3390/healthcare14213610 (PMC13762747; doi:10.3390/healthcare14213610)
Supplement: Supplementary file 1 [file healthcare-14-03610-s001.zip › S1 Supplementary Material.pdf]

Supplementary Material S1

Robustness Re-Calculation: Need and Treatment Intensity

Supplement to: Boeckle, M.; Haid, B.; Patek, F.; Löffler-Stastka, H. Psychotherapeutic Care in Austria 2026–2040: An FTE-Calibrated Simulation of Workforce, Funding and Care Capacity. Healthcare (submitted).

S1.1. Purpose

This supplementary calculation tests the stability of the FTE-calibrated model by modelling need not only at 9% but at 5%, 7.5%, 9%, and 12% of the population, and by varying treatment intensity at 13, 20, 30, and 40 units per patient per year.

S1.2. Calculation Logic

The starting point is the theoretical capacity of 669,121 persons/year at 13 units per patient per year. For higher treatment intensities, capacity is reduced proportionally: capacity(E) = 669,121 × 13/E. Need is calculated as population × need share, with the population operationalized as the 8,942,791 persons entitled to benefits in Austrian social health insurance in the 2022 annual average (Dachverband der Sozialversicherungsträger, Statistisches Handbuch, Chapter 2.1; reference [11] of the manuscript); at 9% this yields a need of 804,851 persons per year. Accessible capacity results from potential capacity × activation rate. The care gap is need minus accessible capacity, bounded below at 0. The full-funding share is defined relative to potential capacity, consistent with the definition used throughout the manuscript.

S1.3. Scenario Assumptions

Scenario	Activation rate	Full-funding share
A Status quo	30%	14.5%
B Partial funding	57%	39.0%
C Full expansion	82%	76.0%

Note: The deterministic robustness calculation anchors the activation modes on the empirically observed 2022 value (30% = 199,250/669,121) and the rounded scenario means of the main simulation (57%; 82%), and the full-funding share on the empirical 2022 anchor (14.5% ≈ 96,500/669,121) and the means of the triangular distributions in Table A2 (39%; 76%). The main Monte-Carlo model draws these parameters from the triangular distributions documented in Table A2 of the manuscript; resulting differences in coverage are on the order of one to two percentage points and do not affect the scenario ranking (cf. Supplementary Material S3, Section S3.8.4).

S1.4. Core Evaluation at 9% Need

Scenario	Units/patient/year	Coverage, accessible (%)	Care gap, accessible (persons)
A Status quo	13	24.9	604,115
B Partial funding	13	47.4	423,452
C Full expansion	13	68.2	256,172
A Status quo	20	16.2	674,373
B Partial funding	20	30.8	556,942
C Full expansion	20	44.3	448,210
A Status quo	30	10.8	717,866
B Partial funding	30	20.5	639,578
C Full expansion	30	29.5	567,090
A Status quo	40	8.1	739,612

Scenario	Units/patient/year	Coverage, accessible (%)	Care gap, accessible (persons)
B Partial funding	40	15.4	680,897
C Full expansion	40	22.2	626,530

S1.5. Interpretation

The robustness calculation shows that the model's message depends strongly on the assumed treatment intensity. At 13 units per patient per year, Scenario C can computationally cover even high need levels to a large extent. At 30 or 40 units per patient per year, theoretical capacity falls markedly; then even a high activation rate no longer suffices in all need scenarios. This makes visible that the central policy message remains stable—activation and funding are central levers—but professionally more intensive psychotherapy additionally requires more capacity, more phase-3 places, and more structural care institutions.

S1.6. Files

Table S1: Robustheitsmatrix_Bedarf_Einheiten_Szenarien.csv — deterministic robustness matrix (48 constellations: 4 need levels × 4 treatment intensities × 3 scenarios). Table S2: MC_Robustheit_Bedarf_Einheiten.csv — Monte-Carlo robustness summary (medians, 80% and 95% prediction intervals for coverage and care gap; n = 20,000 per cell). Table S3: MC_FTE_Kalibriert_AllYears_ANNOTATED.csv — annual medians and prediction intervals of the main simulation, 2026–2040. Table S4: MC_FTE_Kalibriert_Sensitivitaet_within_ANNOTATED.csv — within-scenario Spearman correlations of the model parameters with cumulative follow-on costs 2040. Table S5: MC_FTE_Kalibriert_Sensitivitaet_Zielgroessen_ANNOTATED.csv — within-scenario correlations with costs, accessible capacity and licensed stock (Table 6 of the manuscript). Table S6: MC_FTE_Kalibriert_Masterplaetze_ANNOTATED.csv — Master-intake analysis (Table 7 of the manuscript). Code S1: montecarlo_psychotherapie_fte_annotiert_mit_quellen.py — annotated source code of the main Monte-Carlo model, model version 2.1 (key excerpts in Supplementary Material S3, Section S3.8). Code S2: robustness re-calculation script (need × treatment intensity). All random draws are reproducible via the fixed seed 20260622.

Further files (Sections S1.7–S1.11): Table S9: Table_S9_Stundenanker_Sensitivitaet.csv; Table S10: Table_S10_Intensitaet_dynamische_Kapazitaet.csv; Table S11: Table_S11_Trainee_Mechanismen_Trennung.csv; Table S12: Table_S12_ChronWartezeit_Varianten.csv; Table S13: Table_S13_Hebelzerlegung.csv; Figures S1–S4: Figure_S1_Heatmap_Coverage_A_Status_quo.png, Figure_S2_Heatmap_Coverage_B_Partial_funding.png, Figure_S3_Heatmap_Coverage_C_Full_expansion.png, Figure_S4_Care_gap_9pct_need_by_intensity.png (shown below).

S1.7. Weekly-Hours-Anchor Sensitivity (Table S9)

Because potential capacity scales linearly with the weekly-hours anchor, the full Monte-Carlo simulation (Code S1, seed 20260622, n = 30,000) was re-run with the anchor set to 15.8 and 13.3 treatment hours/week instead of 21, the activity-adjusted weighted means for contracted and for all listed practitioners from the authors' secondary analysis of the individual-level data of the ÖBVP member survey of 2–20 December 2024 (reference [9] of the manuscript; 2,777 respondents, response rate 43.5%). For these estimates the 638 respondents in training were excluded (2,139 listed practitioners, 984 of them contracted); each respondent's weekly time in private practice including administration (category midpoints) was multiplied by the reported share of psychotherapeutic treatment in practice activity, and the sample was post-stratified by iterative proportional fitting to the federal-register margins sex, age band, regional association and contract status (31 December 2023; design effect 1.83, effective n = 1,166). Weighted means: contracted practitioners 21.1 practice hours/week with a treatment share of 75.1%, i.e., 15.8 treatment hours/week (bootstrap 95% interval 15.1–16.4; 400 replicates with the weights re-estimated in each replicate); all listed practitioners 19.1 practice hours/week and 70.9%, i.e., 13.3 treatment hours/week (13.0–13.7; computed per respondent, so the weighted mean of the products need not equal the product of the weighted means). Both values include the administrative component of treatment work and are weighted means, whereas the 21-hour anchor is a published median of treatment hours; neither figure is a correction of the other (Section 5.10

of the manuscript). The capacity triangle per registered therapist (50; 57.31; 68 at 21 h/week) and the trainee module (72.69 persons/year per fully active therapist) are rescaled consistently by h/21; all random draws are identical to the main model. Table S9 reports, per anchor and scenario, the 2040 medians of potential, accessible and fully funded capacity, the care gap (2040 and minimum), the first year of gap closure, and cumulative follow-on costs. Key result: under 15.8 h/week the median gap no longer closes in Scenario B and closes in Scenario C only from 2031; under 13.3 h/week it no longer closes even in Scenario C (minimum ≈ 5,500 persons/year); the 21-hour results are therefore upper bounds on capacity.

S1.8. Treatment Intensity at Dynamically Projected Capacity (Table S10)

Table S10 applies the four treatment intensities (13/20/30/40 units/patient/year) to the dynamically projected accessible capacity (medians) of 2030 and 2040 per scenario, as coverage of a 9% population need (804,851 persons/year). It complements the static robustness matrix (Tables S1–S2, 2023 capacity anchor): in Scenario C, coverage at 13 units/patient/year reaches 104%/113% (2030/2040) but only 45%/49% at 30 units/patient/year.

S1.9. Separation of the Two Trainee Mechanisms (Table S11)

Table S11 isolates the pure accessibility effect of trainee funding: capacity per trainee is held at the unfunded level f_0 while trainee services become financially accessible as in the main model (the f_1 draw is retained unused, so random streams are identical). Scenario B: cumulative follow-on costs €15.7 billion (vs. €13.1 billion main model and €31.0 billion without any trainee funding); the median gap no longer closes completely (minimum ≈ 10,600 persons/year). The complete 2032–2036 closure in Scenario B therefore depends on the funding-induced capacity rise from f_0 toward f_1.

S1.10. Scenario-Independent Chronification and Waiting-Time Variants (Table S12)

Table S12 implements the three variants of Table 1 of the main text as separate runs (Code S1, Section 15): the chronification rate is fixed at the variant's base risk (0.10/0.18/0.28) and the waiting-time cost factor is drawn from a triangular distribution over the variant's range with mid-point mode (1.0; 1.15; 1.3 / 1.0; 1.40; 1.8 / 1.0; 1.75; 2.5), identically in all three scenarios; all policy parameters remain scenario-specific. Care gaps and capacities are unaffected by construction (verified by assertion); cumulative follow-on costs 2040 are reported with 95% uncertainty intervals. The A–C difference ranges from €51.6 billion (conservative) to €76.0 billion (pessimistic), versus €82.1 billion in the main model.

S1.11. Sequential Policy-Lever Decomposition (Table S13)

Table S13 reports the sequential decomposition from Scenario A to Scenario C (Code S1, Section 16): parameter groups are switched cumulatively to their Scenario C distributions under common random numbers — (S1) activation of listed therapists, (S2) insurance funding of trainee services, (S3) training capacity (phase-3 places, completion rate), (S4) clinical-economic parameters. Reported endpoints: median care gap 2040, median accessible capacity 2040, median cumulative follow-on costs 2040, first year of gap closure, and step-wise deltas.

S1.12. Consistency Constraint

Code S1 constrains the drawn full-funding share in each simulation draw to the activation rate of listed therapists (full-funding share ≤ activation; Section 3.3 of the main text), so that fully funded capacity cannot exceed accessible capacity. The constraint acts only on the fully funded capacity and on the within-scenario rank correlations of the full-funding share (Scenario C: Spearman ρ −0.10 with cumulative follow-on costs and +0.14 with accessible capacity; Table 6 of the main text); care gaps, accessible capacity, workforce stocks and follow-on costs do not depend on the full-funding share. The deterministic robustness matrix of Code S2 (Table S1) uses fixed full-funding shares below the respective activation rates (Section S1.3).

Supplementary Figures S1–S4

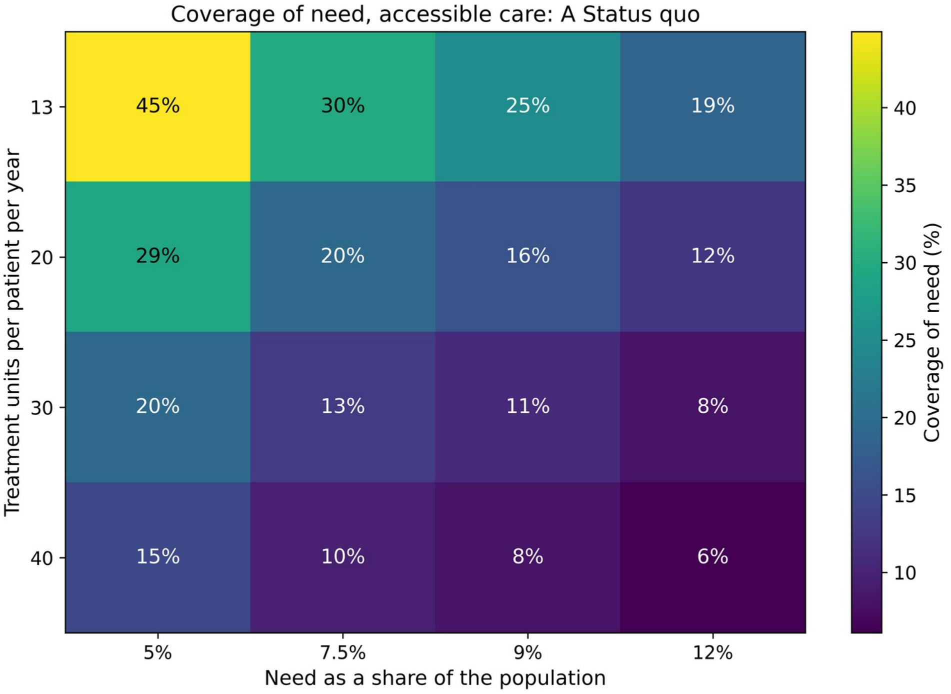

Figure S1. Coverage of need by accessible care in Scenario A (status quo/underfunding) across need levels (5–12%) and treatment intensities (13–40 units/patient/year). Static 2023 capacity anchor (669,121 persons/year at 13 units/patient/year). Source: authors' simulation.

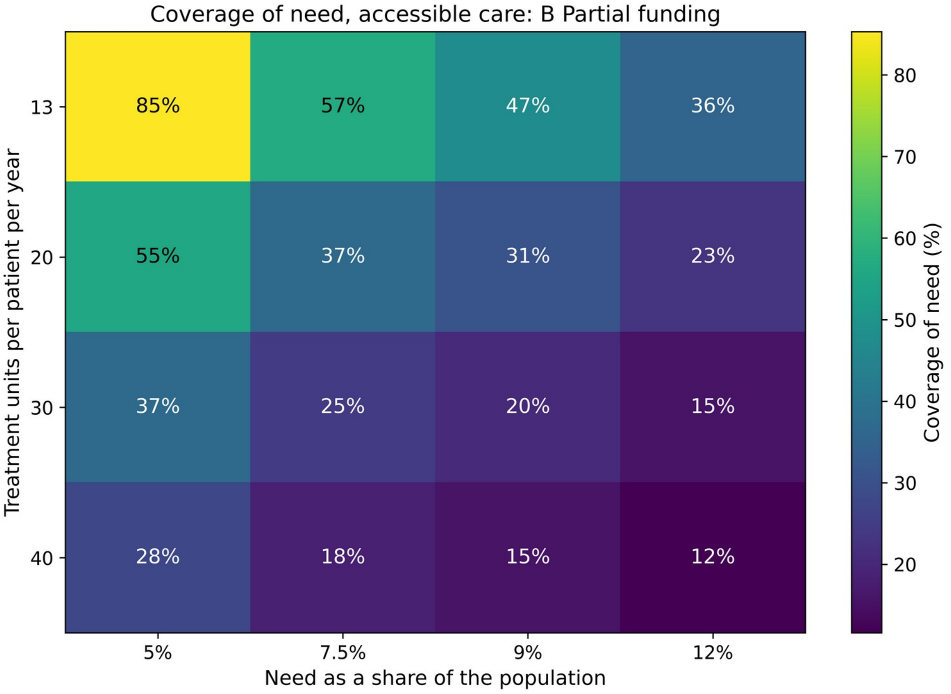

Figure S2. Coverage of need by accessible care in Scenario B (transition/partial funding). Static 2023 capacity anchor. Source: authors' simulation.

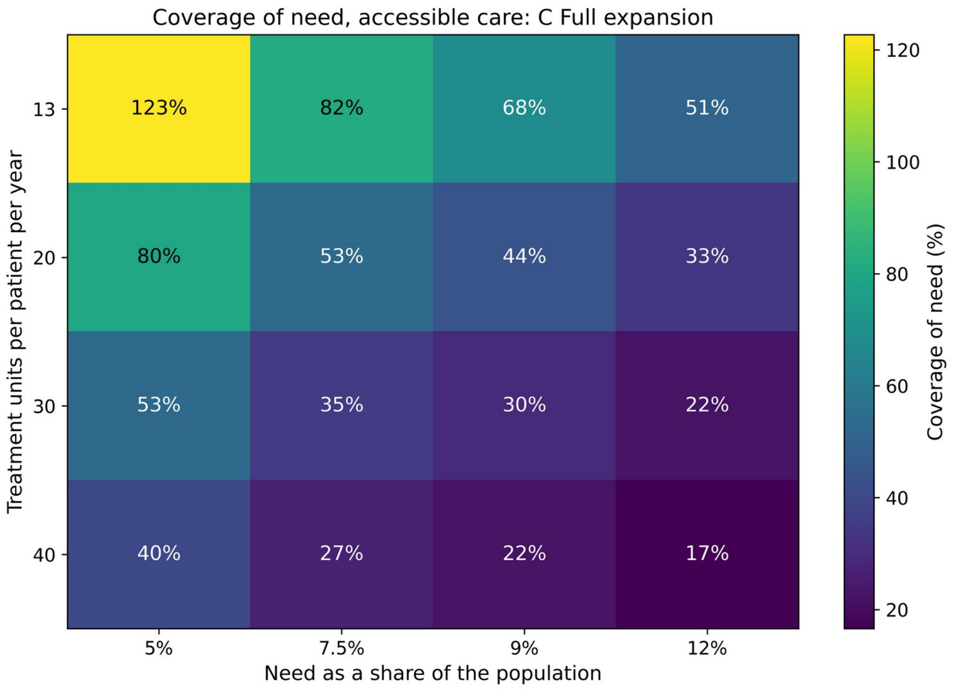

Figure S3. Coverage of need by accessible care in Scenario C (expansion/full funding). Static 2023 capacity anchor. Source: authors' simulation.

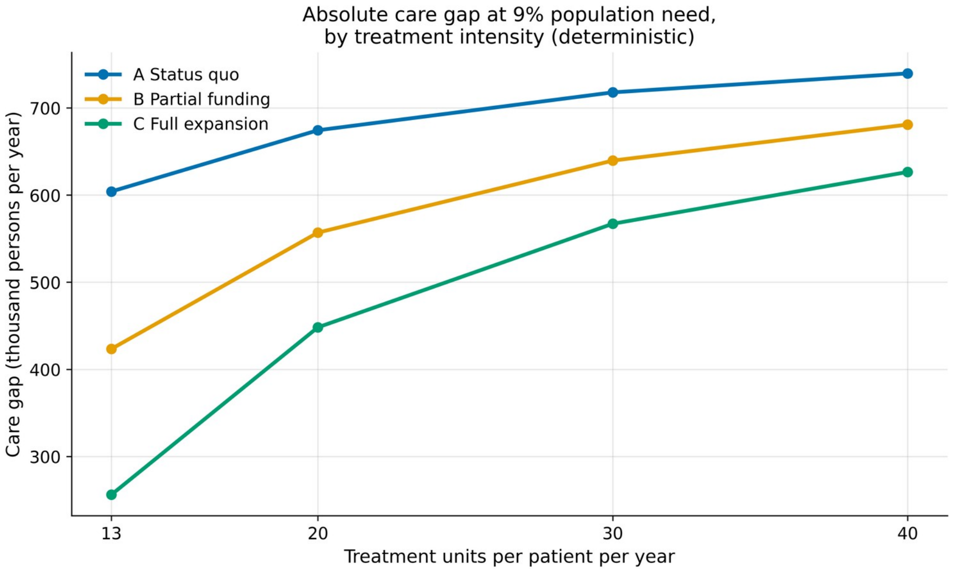

Figure S4. Absolute care gap (insufficiently treated persons per year) at 9% need by treatment intensity and scenario (deterministic calculation, static 2023 capacity anchor). Source: authors' simulation.
