# Supplementary material for: Psychotherapeutic Care in Austria 2026–2040: An FTE-Calibrated Simulation of Workforce, Funding and Care Capacity
Source: Healthcare (Basel). 2026 Oct 25;14(21):3610. doi: 10.3390/healthcare14213610 (PMC13762747; doi:10.3390/healthcare14213610)
Supplement: Supplementary file 1 [file healthcare-14-03610-s001.zip › S2 Supplementary_Material.pdf]

Supplementary Material S2

Stratified Re-Analysis of the ÖBVP Member Survey (December 2024): Treatment Activity of Trainees under Supervision versus Listed Psychotherapists (Table S7)

Supplement to: Boeckle, M.; Haid, B.; Patek, F.; Löffler-Stastka, H. Psychotherapeutic Care in Austria 2026–2040: An FTE-Calibrated Simulation of Workforce, Funding and Care Capacity. Healthcare (submitted). Model version 2.1.

S2.1. Purpose

The simulation treats supervised trainees of the old (i.A.u.S.) and the new training system (PiFALS, psychotherapists in specialist training under teaching supervision) as a capacity line of their own (Section 3.8.2 of the main text). Their caseload and treatment hours are not published in official statistics. This note documents the empirical anchor of the capacity per trainee in the current, unfunded state (parameter f_0; triangular distribution 0.50; 0.60; 0.70 of a fully active therapist): a stratified re-analysis of the authors' member survey of December 2024, whose pooled results are reported in Boeckle et al. (2025) [9]. The survey publication reports the profession as a whole and, for trainees, only their interest in insurance-funded places (96%); the trainee-specific activity figures below are reported here for the first time.

S2.2. Survey and Population

The online survey was conducted by the Austrian Federal Association for Psychotherapy (ÖBVP) from 2 to 20 December 2024 among all members who were either listed in the professional register of the Federal Ministry (BMSGPK) or held the status "in training under supervision" (in Ausbildung unter Supervision, i.A.u.S.). Participation via a personalized link was voluntary, anonymous and possible once; opening the link constituted informed consent; two reminders were sent; the questionnaire consisted of closed questions and took about six minutes [9]. Of 6,388 invited members, 2,777 responded (43.5%): 2,139 listed psychotherapists (45.6% of the 4,689 listed ÖBVP members; 18.3% of all 11,676 listed psychotherapists in 2023) and 638 trainees under supervision (37.6% of the 1,699 ÖBVP trainee members; 22.8% of all 2,799 trainees under supervision in 2023) [9, Table 1]. Respondents are on average younger (50.3 years) than the registered profession as a whole (57.8 years), which the survey publication interprets as a possible bias towards younger and more active members [9].

S2.3. Methods of the Re-Analysis

The individual-level responses were stratified by professional status (listed versus i.A.u.S.) and summarized without weighting. All activity items were asked in categories; category midpoints were used to compute means, medians and ratios: weekly hours of psychotherapeutic activity (including administration) in free practice and, separately, in an institution in 2024 (less than 5 h = 2.5; 5–10 h = 7.5; 11–20 h = 15.5; 21–30 h = 25.5; more than 30 h = 35); number of patients treated in free practice in 2024 (less than 10 = 5; 10–20 = 15; 21–30 = 25.5; 31–40 = 35.5; 41–50 = 45.5; 51–60 = 55.5; 61–70 = 65.5; 71–80 = 75.5; more than 80 = 90); possible expansion of weekly hours among respondents planning to work more in 2025–2030 (up to 5 h = 2.5, up to 10 h = 7.5, up to 15 h = 12.5, up to 20 h = 17.5, up to 25 h = 22.5, more than 25 h = 27.5; category upper bounds 5–30 h as an upper variant). Total weekly hours are the sum of free-practice and institutional hours (respondents without any activity in a channel contribute zero to that channel; respondents without hours in either channel are excluded). Hours and patients in free practice refer to the respondents working in free practice (the items were not asked of the others); institutional hours refer to the respondents employed in an institution. Fees per 50-minute session were asked in €10 categories and are reported as the median category with the interquartile range. Ratios (trainees/listed) are computed on the means. The stage effect (S2.5) stratifies the trainees by the year in which they planned to complete their training and to be listed.

S2.4. Table S7

Table S7. Treatment activity, occupation, expansion plans and fees of trainees under supervision (i.A.u.S.) versus listed psychotherapists, ÖBVP member survey, December 2024 (unweighted; category midpoints).

Indicator	i.A.u.S. (n)	i.A.u.S.	Listed (n)	Listed
Working in free practice (share)	638	96.1%	2139	98.6%
Employed in an institution (share)	638	24.0%	2139	19.4%
Psychotherapy is the sole occupation (share)	638	39.7%	2139	66.9%
Weekly hours in free practice, incl. administration, 2024 (mean)	613	12.2	2108	19.8
Weekly hours in free practice, incl. administration, 2024 (median)	613	7.5	2108	15.5
Weekly hours in an institution, 2024, employed only (mean)	153	18.3	415	18.4
Total weekly hours, free practice + institution, 2024 (mean)	634	16.2	2135	23.2
Total weekly hours, free practice + institution, 2024 (median)	634	15.5	2135	25.5
Patients treated in free practice in 2024 (mean)	613	17.4	2108	34.7
Patients treated in free practice in 2024 (median)	613	15.0	2108	25.5
Planned scope 2025–2030 vs. 2024: more (share)	638	82.0%	2139	29.5%
Planned scope 2025–2030 vs. 2024: unchanged (share)	638	16.8%	2139	56.5%
Planned scope 2025–2030 vs. 2024: less (share)	638	1.3%	2139	14.0%
Possible expansion, weekly hours, among those planning more (mean, category midpoints)	523	12.6	631	7.7
Possible expansion, weekly hours, among those planning more (mean, category upper bounds)	523	15.1	631	10.2
Fee per 50-min session (median category; interquartile range)	613	bis 80 € (bis 70 €–bis 80 €)	2108	bis 100 € (bis 100 €–bis 110 €)
Fee per 50-min session up to €80 (share)	613	78.3%	2108	5.3%
Fee per 50-min session €90–120 (share)	613	21.2%	2108	87.7%

Note: n = respondents with a valid answer to the respective item. Ratios of the means, trainees/listed: weekly hours in free practice 0.61; total weekly hours 0.70; patients per year in free practice 0.50. Interest in fully funded insurance places after listing (asked of trainees only, n = 638): yes 46.6%, only at higher tariffs 49.8%, no 3.6% (reported as 96% interest in [9]). Fee categories: 'bis 80 €' = up to €80 etc. Source: authors' survey data, partly reported in [9]; script survey_table_S7.py.

S2.5. Stage Effect within the Trainee Group

Table S7b. Trainees under supervision by planned year of completion and listing (unweighted; category midpoints).

Planned completion	n	Weekly hours, free practice (mean)	Total weekly hours (mean)	Patients per year, free practice (mean)	Planning more hours (share)
2024/2025	321	14.7	19.6	21.6	78%
2026	230	10.5	14.2	14.4	84%
2027 or later	87	6.8	9.0	8.4	91%

Note: Planned completion year as stated in December 2024; respondents without a valid year are omitted. Treatment activity rises with the stage of training: candidates close to completion treat about twice as many hours and patients as candidates with two or more years to go. In the model this within-training build-up is represented by the lead time to full trainee activity (0.5–2 years) for the new system and, for the old system, by the average over all stages implicit in fo.

S2.6. Derivation of the Model Parameter f_0

Relative to listed psychotherapists, trainees under supervision report 0.61 of the mean weekly hours of psychotherapeutic activity in free practice, including administration (means 12.2 versus 19.8 h; medians 7.5 versus 15.5 h), 0.70 of the mean total weekly hours including institutional work (16.2 versus 23.2 h) and 0.50 of the patients treated per year in free practice (17.4 versus 34.7). The listed respondents' total of 23.2 h per week is of the same order as the empirical FTE anchor of the model (median 21 treatment hours plus 3 documentation hours per week, Dinhof et al. [2]). This total, however, includes institutional work and, within private practice, activities other than treatment (supervision, teaching, self-experience); treatment time in private practice alone is lower (13.3 h per week among all listed practitioners in a weighted secondary analysis of the same survey; Section 5.10 of the main text and Supplementary Material S1.7). The model therefore uses the survey only for ratios between trainees and listed psychotherapists measured with the same items, not for absolute levels. Because the model's capacity is defined in treatment hours (72.69 persons per year = 945 h/13 units), the hours-based ratios (0.61–0.70) are the primary anchor and the patient-based ratio (0.50) the lower bound; the triangular distribution f_0 ~ (0.50; 0.60; 0.70) spans this range with its mode in the middle. The same value is applied to phase-3 trainees of the new system (PiFALS), for whom no data exist yet; their training is planned as 50% employment with the remaining time freely chosen, which is compatible with the observed part-time pattern. Only 40% of the trainees practise psychotherapy as their sole occupation (listed: 67%), 82% plan to expand their hours by 2030 (mean +12.6 h per week with category midpoints, +15.1 h with upper bounds), and 47% would take insurance-funded places unconditionally, a further 50% at higher tariffs. These figures motivate the funding-dependent formulation of the main model, in which the capacity per trainee rises from f_0 towards f_1 (0.80; 0.90; 1.00; expert assumption) as trainee services become insurance-funded, with a one-year lag for practice build-up. Fees are lower among trainees (median category up to €80; 78% charge €80 or less) than among listed psychotherapists (median category up to €100; 88% charge €90–120), which indicates that trainee services are currently priced as a discounted, privately paid service.

S2.7. Limitations

The survey is a single cross-section of ÖBVP members with a 43.5% response rate; it is unweighted, so the trainee group (22.8% of all trainees in 2023) and the listed group (18.3% of all listed psychotherapists) may differ from their populations, in particular towards younger and more active members [9]. All activity items are categorical; midpoints introduce a rounding error of a few percent, and the open upper categories (more than 30 h; more than 80 patients) were assigned conservative values. Hours "including administration" overstate treatment hours slightly and equally in both groups, so the ratios are affected less than the levels. Expansion plans are stated intentions, not observed behaviour. Trainee-specific figures refer to the old system (i.A.u.S.); their transfer to phase-3 trainees of the new system is an assumption. The individual-level responses are not deposited; the aggregate statistics in Table S7 and the analysis script are provided.

S2.8. Files

Table S7: Table_S7_survey_reanalysis.csv (this table in machine-readable form); Table_S7_stats.json (all underlying statistics incl. the stage effect and interest items); survey_table_S7.py (analysis script; reads the raw survey export, which is not deposited).
