# Supplementary material for: Psychotherapeutic Care in Austria 2026–2040: An FTE-Calibrated Simulation of Workforce, Funding and Care Capacity
Source: Healthcare (Basel). 2026 Oct 25;14(21):3610. doi: 10.3390/healthcare14213610 (PMC13762747; doi:10.3390/healthcare14213610)
Supplement: Supplementary file 1 [file healthcare-14-03610-s001.zip › S3 Supplementary_Material_Technical_Documentation.pdf]

Supplementary Material S3. Technical Documentation of the Monte-Carlo Model

Supplement to: Psychotherapeutic Care in Austria 2026–2040: An FTE-Calibrated Simulation of Workforce, Funding and Care Capacity (Healthcare).

This supplement documents the Monte-Carlo model at the depth required for reproduction and critical review: model architecture (S3.1), empirical data anchors with source attribution (S3.2), reproducibility and implementation (S3.3), detailed methodological limits (S3.4), verification and calibration checks (S3.5), the operationalization of the activation rate (S3.6), a numerical summary of the robustness matrix (S3.7) and annotated code excerpts (S3.8). The core model formulas and the scenario-specific parameter distributions with their evidential status are given in Appendix A of the article (Tables A1 and A2). Section, table and figure numbers without the prefix S3 or D, and reference numbers in square brackets, refer to the article. The complete annotated code is provided as Code S1 (main model) and Code S2 (robustness re-calculation); all random draws are reproducible via the fixed seed 20260622.

S3.1. Overview of the Model Architecture

The model is implemented as a discrete annual update from 2026 to 2040 (T = 15 years), with 30,000 Monte Carlo iterations per scenario. Each simulation run draws all stochastic parameters from their triangular distributions during initialization; the annual loop then updates stock, pipeline, capacities, care gap, and costs consistently over the whole projection horizon. The calculation sequence in each year is fixed: (1) update of the old Fachspezifikum pool with admissions (until 2030), completions and drop-outs, accelerated completion 2031–2038 and derivation of the i.A.u.S. stock; (2) phase-3 completions (licensures) of the new system; (3) new phase-3 entrants from the Master cohorts (two years after entry) and direct entrants, first-in-first-out allocation of free phase-3 places; (4) treating trainee stock (after the lead time; waiting candidates at 50%) and trainee capacity with the funding-dependent capacity factor ($f_0 \rightarrow f_1$, one year behind the funding ramp); (5) computation of retirements and other exits and workforce update PT(t+1); (6) potential capacity from therapists and trainees; (7) accessible capacity from the activation rate (therapists) and the phased-in insurance funding of trainee services; (8) computation of the care gap; (9) computation of annual and cumulative follow-on costs.

S3.2. Empirical Data Anchors with Source Attribution (Q1–Q5)

The code header documents the model parameters via five source groups (Q1–Q5), enabling direct tracing of each model anchor to its primary source (Table D1).

Table D1. Empirical data anchors with source attribution.

Q	Data anchor	Source
Q1	11,676 psychotherapists (31 December 2023); of whom 8,523 < 65 years, 3,159 ≥ 65 years; median 21 h/week plus 3 h documentation; 45 weeks/year; 13 units/patient/year; theoretical total capacity ≈ 669,121 persons/year; 199,250 partially/fully funded and 96,500 fully funded persons reached in 2022; need/treatment willingness ≈ 9%.	[2]
Q2	Social-insurance expenditure 2022: ~€135 million; 370,389 persons in a broad psychosocial count; 96,500 fully funded psychotherapy in the narrow sense; expansion by a factor of 3–4 required for need coverage.	[3]
Q3	Training statistics 2025 (reference date 1 June 2025; model anchor): 5,601 in the Fachspezifikum, of whom 3,238 (57.8%) with the status 'in training under supervision'; 929 admissions; 624 completions; 91 drop-outs (drop-out share of throughput ≈ 12.7%); Master programme in psychotherapy offered from the winter semester 2026/27; register listing of trainees from 1 October 2026 with insurance-benefit eligibility. For reference, training statistics 2023: 5,021 in the Fachspezifikum; 875 admissions; 514 completions; 85 drop-outs.	[14,13]
Q4	3.5% care target: 13,125 therapists; 5% care target: 18,750 therapists; personnel supply 2040 between 10,660 and 14,329 depending on retirement assumption.	[4]
Q5	Average age of the registered profession 57.8 years; 3,719 persons (31.9%) aged 50–65 and 3,156 (27.0%) aged 66 or older (Table 2 therein, based on BMSGPK data for 2023); 2,799 persons in specialist training under teaching supervision in 2023 (Table 1 therein); 27–40% age-related exits/reductions by 2040; 83% of therapists without a benefits-in-kind contract and 96% of supervised trainees express interest in insurance-funded places (tariff-dependent). Survey population: 6,388 ÖBVP members invited, 2,777 responses (43.5%), of	[9]

Q	Data anchor	Source
	whom 2,139 listed psychotherapists and 638 trainees under supervision (37.6% of the 1,699 ÖBVP trainees; 22.8% of all 2,799 trainees in 2023); the trainee-specific treatment hours, patients, occupation and fees are not reported in [9] and are re-analysed from the authors' survey data in Table S7.	

Note: Persons entitled to benefits in Austrian social health insurance, 2022 annual average: 8,942,791 (operational reference quantity for need modeling) [11]. The denominator is held constant over the projection horizon; the 2023 and 2024 annual averages (9,020,191 and 9,063,328) are about 0.9% and 1.4% higher, so a dynamic denominator would raise modeled need—and hence the care gap—by roughly one percentage point without altering the scenario ranking.

S3.3. Reproducibility and Implementation

The simulation is implemented in Python 3 and uses NumPy for random number generation and vectorization, Pandas for result aggregation, SciPy for rank correlations, and Matplotlib for graphics. All random draws are reproducible through a fixed seed (np.random.seed(20260622); n_sim = 30,000; years 2026–2040). Code S1 contains the consistency constraint (full-funding share ≤ activation rate), Sections 15–17 (Tables S12, S13 and S10) and two run-time switches for the weekly-hours anchor (Table S9) and the separation of the trainee mechanisms (Table S11); without switches it reproduces the main model, and all additional analyses use the same random streams. Results are exported as CSV files: (1) MC_FTE_Kalibriert_AllYears_ANNOTATED.csv with annual medians and percentile intervals per scenario, including the trainee stocks and the waiting queue; (2) MC_FTE_Kalibriert_Summary_2040_ANNOTATED.csv with final-year results; (3) MC_FTE_Kalibriert_ParamsSample_ANNOTATED.csv with a sample of drawn parameters and final-year results (n = 10,000 rows); (4) MC_FTE_Kalibriert_Sensitivitaet_within_ANNOTATED.csv with the within-scenario Spearman correlations with costs; (5) MC_FTE_Kalibriert_Sensitivitaet_Zielgroessen_ANNOTATED.csv with the correlations for costs, accessible capacity and licensed stock (Table 6); (6) MC_FTE_Kalibriert_Masterplaetze_ANNOTATED.csv with the Master-intake analysis (Table 7); (7) MC_FTE_Kalibriert_Traineekapazitaet_ANNOTATED.csv with the trainee-capacity robustness analysis (Table 8). All graphics are exported at 600 dpi. The code file (montecarlo_psychotherapie_fte_annotiert_mit_quellen.py, about 1,030 lines) is available as a supplement and contains complete documentation of the empirical anchors with explicit source attribution (Q1–Q5) and a version note in its header. The deposited code is model version 2.1, which adds the trainee capacity line, the Master-entry cohorts, the phase-3 queue, and the 2025 training anchors (version 2) and the funding-dependent capacity per trainee (version 2.1) to the first version; the first version—which treated trainees as a small, phase-out-driven side channel—is retained in the repository as a versioned record.

S3.4. Methodological Limits in Detail

Beyond the general limitations in Section 5.10, the following specific limits are relevant for interpreting the numerical results. **Chronification curve:** despite consistent qualitative evidence, an empirically robust Austrian risk curve for chronification after 1, 3, 6, or 12 months of waiting is lacking; the waiting-time–chronification relationship is therefore carried as a sensitivity parameter with three variants (Table 1); empirical calibration is urgently needed once longitudinal data become available. **Cost parameters as scenario assumptions:** the cost components (base cost €5,000, chronification excess €10,000, productivity cost €3,000/year; modes) are scenario assumptions whose absolute values remain to be validated with Austrian data on sick leave, earnings losses, invalidity pensions, and healthcare costs; the qualitative validity of the scenario ranking is robust, while absolute euro amounts are order-of-magnitude indicators, not precise forecasts. **Trainee capacity:** the number of persons in training under supervision is published (3,238 on 1 June 2025 [14]), but their caseload and weekly treatment hours are not published; the unfunded level (0.50; 0.60; 0.70 of a fully active therapist) is anchored on a stratified re-analysis of the authors' member survey (Table S7: ratio of mean weekly hours 0.61–0.70, of patients 0.50), while the fully funded level (0.80; 0.90; 1.00) is an expert assumption; their small within-scenario correlations with costs ($|\rho| \leq 0.03$) do not mean a small effect—the trainee line supplies 18% of potential capacity in 2026 and up to 30% in 2032, and its level determines the year in which Scenario B closes the gap (Table 8); the assumption therefore belongs to the first calibration priorities. **Retirement dynamics:** the age structure of the profession is empirically known (Q5: 27% ≥ 66 years), but individual exit ages and the reduction of weekly hours by age group are not; the modeling with a 3.5% base rate growing linearly to up to 5.5% spans the plausible range; the

moderate cost sensitivity of this parameter (ρ = +0.06 to +0.15) and its dominant role for the licensed stock (ρ ≈ −0.8) are discussed in Section 5.5.

S3.5. Model Verification and Calibration Checks Against 2022/2023 Workforce Data and 2025 Training Data

A validation section is methodologically central for care-economic simulation models. Four verification and calibration steps are documented here: (a) face validity (verification), (b) a calibration consistency check against the 2022/2023 workforce and funding data and the 2025 training statistics (reproducing the empirical starting values is calibration rather than external validation), (c) extreme-scenario testing (verification), and (d) reproducibility checking (verification).

Table D2. Calibration consistency check against empirical values 2022–2025.

Quantity	Empirical value 2022–2025	Model (starting value)	Status
Licensed therapists (31 December 2023)	11,676	11,676	exact
Theoretical capacity at 13 units	669,121	669,121	exact
Partially/fully funded reached, 2022	199,250	199,250 (activation 0.298)	exact
Fully funded reached, 2022	96,500	96,500 (full funding 0.144)	exact
Supervised trainees, old system (1 June 2025)	3,238	3,238 (initial value)	exact
Persons in the Fachspezifikum (2023 → 2025 → model 2026)	5,021 → 5,601	5,835 (2026, median)	trend consistent (+4–6%/year)
Admissions, Fachspezifikum (2024/25)	929	950 (mode; 850–1,100)	plausible (2023/24: 1,058)
Completions, Fachspezifikum (2024/25)	624	637 (2026, median)	+2%, plausible
Persons in the Fachspezifikum (1 June 2025)	5,601	5,601 (initial value)	exact
Drop-out share of annual throughput (2024/25)	≈ 12.7% (91/715)	12.7% (model value)	exact

Note: The exact matches are not validation results in the narrow sense but calibration commitments—the model was constructed to reproduce the empirical starting values. Two consistency checks go beyond calibration. First, the 2023 training statistics [13] and the 2025 statistics [14] bracket a two-year interval in which the Fachspezifikum stock grew from 5,021 to 5,601 (+5.6% per year), admissions from 875 to 929 (with a peak of 1,058 in 2023/24) and completions from 514 to 624; the model's admission range (850–1,100), turnover time (8–10 years) and first-year completions (637) reproduce this trend. Second, the 2040 licensed stock of Scenario A (15,333) lies above the 10,660–14,329 range of the GÖG projection [4], which did not contain the accelerated completion of the old pathway required by § 60 PThG 2024 nor the Master-entry route of the new system. Validation in the narrow sense would require testing predicted 2026/2027 values against incoming empirical data; this can only be done as data become available.

We checked face validity by discussing the model architecture with representatives from care research, training statistics, and professional policy. Key plausibility findings: (a) the three-level capacity logic (potential/accessible/fully funded) matches the actual care structure in Austria; (b) the four-to-seven-year latency of the Master route (two years of Master studies plus two to five years of phase 3) is consistent with the legally defined training structure; (c) the 3.5% retirement assumption matches the demographic structure (27% over 65). **Extreme-scenario testing:** at an activation rate of 0.00, the care gap equals full need (~805,000 persons/year)—confirmed; at 1.00, the gap equals need minus potential capacity—confirmed (negative gaps correctly bounded at 0 at 13 units; a substantial gap remains at 30 units, i.e., capacity shortage); at a retirement rate of 0.00, the workforce grows only through completions—confirmed; at 0.10, a substantial workforce loss occurs (in Scenario A the stock falls below 5,000 by 2040)—confirmed. **Limits of these checks:** they show that the model reproduces the empirical starting data and behaves plausibly under extreme conditions; genuinely predictive validation is possible only from 2026/2027 onward when the first reform data become available. Until then, the model counts as a scenario model, not a forecasting instrument.

S3.6. Activation-Rate Transparency: Operationalization of the Scenarios

The activation rate—the share of potential capacity translated into accessible care—is the dominant model parameter. Table D3 makes the activation assumptions behind the scenarios transparent and assigns each a political-structural realization scenario.

Table D3. Activation rates and their political-structural interpretation.

Activation rate	Model assignment	Empirical reference	Political-structural realization condition
0%	theoretical lower bound	—	No publicly funded or outpatient-accessible psychotherapy; purely private market.
30%	Scenario A (actual, 2022)	199,250/669,121 = 0.298	Current Austrian care structure: limited insurance quotas, regionally unequal clinic density, high self-payment share.
50–60%	Scenario B	model estimate	Stepwise clinic expansion, partial trainee funding, moderate insurance expansion; comparable to the German needs-planning level.
80–85%	Scenario C	model estimate; ~Switzerland	Substantial insurance expansion, nationwide clinic structure, full trainee funding; comparable to the Swiss prescription model.
100%	theoretical upper bound	—	Hypothetical maximum: every practising therapist fully integrated into the public/partially funded system; not reached in any real system to date.

Note: The assignment is heuristic and serves transparency. The exact activation rate achievable by a given policy measure is an empirical question that can only be evaluated ex post.

S3.7. Robustness Matrix: Numerical Summary

Table D4 summarizes the deterministic coverage (%) across selected constellations of the robustness analysis. Values ≥ 100% indicate full need coverage.

Table D4. Coverage (%) across robustness constellations—Scenarios A/B/C (selection).

Need	Intensity	A: status quo	B: partial funding	C: full expansion	Difference C−A
5%	13 units	45%	85%	**123%**	78 pp
7.5%	13 units	30%	57%	82%	52 pp
**9%**	**13 units**	25%	47%	68%	43 pp
12%	13 units	19%	36%	51%	32 pp
5%	20 units	29%	55%	80%	51 pp
**9%**	**20 units**	16%	31%	44%	28 pp
5%	30 units	19%	37%	53%	34 pp
**9%**	**30 units**	11%	21%	**30%**	19 pp
12%	30 units	8%	15%	22%	14 pp
**9%**	**40 units**	8%	15%	22%	14 pp

Note: Selection of 10 of the 48 robustness constellations. The complete matrix is available as a CSV supplement (Table S1). pp = percentage points. Bold rows mark the constellations central to care policy.

The robustness matrix shows three structural findings. First, the absolute effectiveness of the scenario difference (column 'difference C−A') declines with rising treatment intensity—at 30 units/year it is only 14–19 percentage points versus 32–78 percentage points at 13 units. Second, even Scenario C covers only 30% and 22% of need at 30 and 40 units, respectively, at 9% need. Third, the qualitative scenario ranking (A < B < C) remains stable across all constellations—that is, the model’s central health-policy message remains robust while its quantitative dimension shifts substantially.

S3.8. Annotated Code Excerpts

The following excerpts reproduce the methodologically central sections of the annotated simulation source code (Code S1: montecarlo_psychotherapie_fte_annotiert_mit_quellen.py, model version 2.1, shown without its run-time switches; original German comments retained; in Section S3.8.2 the splitting of phase-3 batches into integer durations and the result bookkeeping are omitted for readability). The complete scripts for the main model (Code S1) and the robustness re-calculation (Code S2) are provided as supplementary materials; all random draws are reproducible via the fixed seed 20260622.

S3.8.1. Reproducibility Settings and FTE/Capacity Anchors (Code S1)

```
np.random.seed(20260622)          # Reproduzierbarkeit der Monte-Carlo-Ziehungen
n_sim = 30000                     # Anzahl der Monte-Carlo-Laeufe
years = np.arange(2026, 2041)     # Projektionszeitraum inklusive 2040

population = 8_942_791            # Anspruchsberechtigte in der SV 2022
baseline_pt = 11_676              # Berufsausuebende PT laut Liste 31.12.2023
fte_hours_week = 21               # Median Behandlungsstunden/Woche (Dinhof et al. 2025)
working_weeks = 45
hours_year = fte_hours_week * working_weeks        # 21 * 45 = 945 h/Jahr
units_per_patient_actual = 13                      # Ist-Wert Einheiten/Patient:in/Jahr
capacity_per_under65 = hours_year / units_per_patient_actual   # 72,69 Personen/Jahr

potential_capacity_anchor = 669_121                # theoretische Gesamtkapazitaet
current_partial_full_funded = 199_250              # teil-/vollfinanziert erreicht 2022
current_fully_funded = 96_500                      # vollfinanziert erreicht 2022
current_funding_activation = 199_250 / 669_121     # = 0,298
current_full_funding_activation = 96_500 / 669_121 # = 0,144
initial_fachspezifikum = 5_601             # Fachspezifikum am 1.6.2025 (Ausbildungsstatistik 2025)
initial_iaus = 3_238                       # davon in Ausbildung unter Supervision (57,8 %)
trainee_capacity_f0 = tri(0.50, 0.60, 0.70)   # Kapazitaet pro Trainee, unfinanziert (Tab. S7)
trainee_capacity_f1 = tri(0.80, 0.90, 1.00)   # bei voller Kassenfinanzierung (Expert:innenannahme)
master_intake = 500 + tri(250, 500, 750)   # Master-Plaetze/Jahr ab WS 2026/27 (oeffentlich + privat)
phase3_duration = tri(2.0, 3.0, 5.0); phase3_lag = tri(0.5, 1.0, 2.0)   # Phase-3-Dauer, Anlaufzeit
```

S3.8.2. Annual Workforce, Trainee and Capacity Loop (Code S1, Simplified Excerpt)

```
full_funding_share = np.minimum(full_funding_share, funding_activation)  # v8: Constraint
for i, yr in enumerate(years):
    # 9.1 Altes System: Aufnahmen bis 2030, Abschluesse, Abschlussfrist 2038 (§ 60)
    entries = old_entries if yr <= 2030 else 0
    old_pool = old_pool + entries
    throughput = old_pool / old_duration            # Umschlagsregel (ca. 9 Jahre)
    if yr > 2030:                                   # beschleunigte Abschluesse bis 2038
        throughput = np.maximum(throughput, old_pool / max(2038 - yr + 1, 1))
    old_grads = throughput * (1 - baseline_dropout) # Abbrueche 12,7 % des Durchsatzes
    old_pool = np.maximum(old_pool - throughput, 0)
    if yr > 2038: old_grads = 0; old_pool = 0
    share = 0.578 if yr <= 2030 else min(1.0, 0.578 + 0.422 * (yr - 2030) / 3)
    iaus_pool = share * old_pool                    # i.A.u.S.-Anteil am Fachspezifikum

    # 9.2 Phase-3-Abschluesse (Approbationen) der belegten Plaetze
    due = [b for b in active if i - b['admission'] >= b['duration']]
    new_grads = sum(b['amount'] for b in due)
    active = [b for b in active if b not in due]

    # 9.3 Zugaenge zur Phase 3: Master-Abschluesse (2 Jahre) + § 10-Direkteinstieg
    master_cohorts.append(master_intake)
    entrants = direct_entrants
    if i >= 2: entrants = entrants + master_cohorts[i - 2] * new_completion
    queue.append({'eligibility': i, 'amount': entrants})

    # 9.4 FIFO-Zuteilung: Plaetze/Jahr x Phase-3-Dauer = gleichzeitige Plaetze
    slots = phase3_capacity * phase3_duration
    free = np.maximum(slots - sum(b['amount'] for b in active), 0)
    for c in queue:                                 # aelteste Kohorte zuerst
        take = np.minimum(c['amount'], free); free -= take; c['amount'] -= take
        active.append({'admission': i, 'eligibility': c['eligibility'],
                       'duration': phase3_duration, 'amount': take})

    # 9.5 Trainee-Kapazitaet: Anlaufzeit ab Phase-3-Beginn; Wartende 50 %; Faktor f0 -> f1
    treating = sum(b['amount'] for b in active if i - b['eligibility'] >= phase3_lag)
    waiting = sum(c['amount'] for c in queue if i - c['eligibility'] >= phase3_lag)
    iful_treating = treating + 0.5 * waiting
    ramp = lambda t: min(max((t - 2026) * 0.25, 0), 1)   # Einschleichpfad 2027-2030
    f = trainee_capacity_f0 + (trainee_capacity_f1 - trainee_capacity_f0) \
        * iaus_financed_factor * ramp(yr - 1)      # Kapazitaet 1 Jahr hinter Finanzierung
    trainee_potential = (iaus_pool + iful_treating) * f * capacity_per_under65

    # 9.6-9.7 Pensionierungen, Austritte, Workforce-Update
    retire_rate = retirement_base + retirement_growth * (i / (T - 1))
```

```
    pt = np.maximum(pt + old_grads + new_grads - pt * retire_rate - pt * other_exit, 0)

    # 9.8-9.10 Drei Kapazitaetsstufen
    registered_capacity = pt * capacity_per_registered_pt
    pot_capacity = registered_capacity + trainee_potential           # Stufe 1
    trainee_activated = trainee_potential * iaus_financed_factor * ramp(yr)
    accessible_capacity = (registered_capacity * funding_activation  # Stufe 2
                           + trainee_activated)
    full_share = np.minimum(full_funding_share / funding_activation, 1)
    fully_funded_capacity = (registered_capacity * full_funding_share  # Stufe 3
                             + trainee_activated * full_share)

    # 9.11 Versorgungsluecke
    care_gap = np.maximum(need_persons - accessible_capacity, 0)
```

S3.8.3. Follow-On Cost Calculation (Code S1)

```
    # 9.9 Folgekosten als Sensitivitaetsannahme
    cost = (
        care_gap * base_unmet_cost * wait_cost_factor      # Wartezeitkosten
        + care_gap * chronic_rate * chronic_extra_cost     # Chronifizierungskosten
        + care_gap * productivity_cost                     # Produktivitaetsverluste
    ) / 1e6   # Ausgabe in Mio. Euro

    # Wichtig: Die absoluten Kostenparameter sind Sensitivitaetsannahmen,
    # weil keine oesterreichische Quelle eine belastbare Chronifizierungs-
    # und Kostenkurve nach Wartezeitmonaten liefert.
```

S3.8.4. Capacity Scaling of the Robustness Analysis (Code S2)

```
# Kapazitaet skaliert invers proportional zur Behandlungsintensitaet:
# Kapazitaet(E) = Kapazitaet_bei_13_Einheiten * 13 / E
for units in [13, 20, 30, 40]:
    potential_capacity = 669_121 * (13 / units)
    for need_rate in [0.05, 0.075, 0.09, 0.12]:
        need_persons = population * need_rate
        for scen, p in scenarios.items():      # A: 0.30 / B: 0.57 / C: 0.82 Aktivierung
            accessible_capacity = potential_capacity * p['activation']
            gap_accessible = max(0, need_persons - accessible_capacity)
            coverage_accessible = accessible_capacity / need_persons
```

The Monte-Carlo component of the robustness analysis (20,000 iterations per cell) varies the activation and full-funding shares as triangular distributions around the scenario modes (A: 0.25/0.30/0.42; B: 0.45/0.57/0.72; C: 0.68/0.82/0.95) and reports medians with 80% and 95% prediction intervals (Figure 7). The robustness re-calculation anchors the activation modes on the empirically observed 2022 value (0.30 = 199,250/669,121) and on rounded scenario means of the main simulation (0.57; 0.82; Table 5 reports 33%/58%/82% as ratios of the 2040 medians), whereas the main model draws from the modes documented in Table A2 (0.32; 0.58; 0.82); the resulting differences in coverage are on the order of one to two percentage points and do not affect the scenario ranking.
